# Proceedings of the Virtual 3^rd^ UK Implementation Science Research Conference: Virtual conference. 16 and 17 July 2020

**DOI:** 10.1186/s13012-020-01062-3

**Published:** 2020-12-17

**Authors:** Noura Bawab, Joanna C. Moullin, Olivier Bugnon, Clémence Perraudin, April Morrow, Priscilla Chan, Emily Hogden, Natalie Taylor, Mark Pearson, Daniele Carrieri, Karen Mattick, Chrysanthi Papoutsi, Simon Briscoe, Geoff Wong, Mark Jackson, Alita Rushton, Kai Elmas, Jack Bell, Agnes Binagwaho, Miriam F. Frisch, Jovial Thomas Ntawukuriryayo, Dieudonné Nkurunziza, Kelechi Udoh, Amy VanderZanden, Laura Drown, Lisa R. Hirschhorn, N. Seward, C. Hanlon, N. Sevdalis, Mike Hurley, Sally Irwin, Jo Erwin, Fay Sibley, Amber Gibney, Andrea Carter, M. Hurley, M. Connelly, H. Sheldon, A. Gibney, R. Hallett, A. Carter, N. Seward, C. Hanlon, T. Colbourn, J. Murdoch, M. Prince, S. Venkatapuram, N. Sevdalis, Chelsea Coumoundouros, Erika Mårtensson, Giulia Ferraris, Louise von Essen, Robbert Sanderman, Joanne Woodford, W. Slemming, R. Drysdale, T. Makusha, L. Richter, Pallari Elena, Kristina Medlinskiene, Justine Tomlinson, Iuri Marques, Susan Richardson, Katherine Striling, Duncan Petty, Humma Andleeb, Aislinn Bergin, Dan Robotham, Sue Brown, Jennifer Martin, Tayana Soukup, Louise Hull, Ioannis Bakolis, Andy Healey, Dulmini Kariyawasam, Augustin Brooks, Simon Heller, Stephanie Amiel, Nick Sevdalis, People with Diabetes Group, Tayana Soukup, Louise Hull, Ioannis Bakolis, Andy Healey, Dulmini Kariyawasam, Augustin Brooks, Simon Heller, Stephanie Amiel, Nick Sevdalis, People with Diabetes Group, Zuhur Balayah, Zarnie Khadjesari, Aoife Keohane, Wilson To, James S. A. Green, Nick Sevdalis, Hossai Gul, Janet Long, Stephani Best, Frances Rapport, Jeffrey Braithwaite, Shalini Ahuja, Gregory Godwin, Gabriel Birgand, Andrew Leather, Sanjeev Singh, V. Pranav, Nathan Peiffer-Smadja, Esmita Charani, Alison Holmes, Nick Sevdalis, on behalf of co-investigators of ASPIRES, Shalini Ahuja, Nathan Peiffer-Smadja, Kimberly Peven, Michelle White, Sanjeev Singh, Marc Mendelson, Alison Holmes, Andrew Leather, Gabriel Birgand, Nick Sevdalis, ASPIRES study coinvestigators, Jackie Dwane, Sean Redmond, Eoin O’Meara Daly, Caitlin Lewis, Julia E. Moore, Sobia Khan, Julia E. Moore, Sobia Khan, Alexandra Ridout, Venetia Goodhart, Sophie Bright, Sattu Issa, Betty Sam, Jane Sandall, Andrew Shennan, Carlos Alberto dos Santos Treichel, Ioannis Bakolis, Rosana Teresa Onocko Campos, Alice Coffey, Helen Flanagan, Martina O’Reilly, Valerie O’Reilly, Pauline Meskell, Maria Bailey, Eileen Carey, Jane O’Doherty, Cathy Payne, Karen Charnley, Dennis H. Li, Nanette Benbow, J. D. Smith, Juan Villamar, Brennan Keiser, Melissa Mongrella, Thomas Remble, Brian Mustanski, Celia Laur, Ann Marie Corrado, Jeremy Grimshaw, Noah Ivers, N. Benbow, K. Macapagal, J. Jones, K. Madkins, J. D. Smith, D. H. Li, B. Mustanski, Logan Manikam, Shereen Allaham, Michelle Heys, Clare Llewellyn, Neha Batura, Andrew Hayward, Yasmin Bou Karim, Jenny Gilmour, Kelley Webb-Martin, Carol Irish, Chanel Edwards, Monica Lakhanpaul, Paulina Daw, Jet Veldhuijzen van Zanten, Alexander Harrison, Hasnain Dalal, Rod S. Taylor, Patrick J. Doherty, Sinead T. J. McDonagh, Colin J. Greaves, Michelle C. White, Andrew J. M. Leather, Nick Sevdalis, Andy Healey, Ben Grodzinski, Harry Bestwick, Faheem Bhatti, Rory Durham, Maaz Khan, Celine Partha-Sarathi, Jye Quan Teh, Oliver Mowforth, Benjamin M. Davies, On behalf of AO Spine RECODE-DCM Consortia, Michael Sykes, Richard Thomson, Niina Kolehmainen, Louise Allan, Tracy Finch, S. Hogervorst, M. C. Adriaanse, H. E. Brandt, M. Vervloet, L. van Dijk, J. G. Hugtenburg, Nataliya Brima, Nick Sevdalis, T. B. Kamara, H. Wurie, K. Daoh, B. Deen, Justine Davies, Andrew Leather, Jennifer Shuldiner, Nida Shah, Ann Marie Corrado, Paul C. Nathan, Noah Ivers, Susan Calnan, Caragh Flannery, Sheena McHugh, Zarnie Khadjesari, Tracey Brown, Alex Ramsey, Henry Goodfellow, Sherine El-Toukhy, Lorien Abroms, Helena Jopling, Michael Amato, Magdalena Jurczuk, Posy Bidwell, Daniel Wolstenholme, Louise Silverton, Jan Van Der Meulen, Nick Sevdalis, Ipek Gurol-Urganci, Ranee Thakar, Andreas Xyrichis, Katerina Iliopoulou, Jessica McCluskey, Patricia Donnelly, Sarah Brady, Sue Franklin, Carol-Anne Murphy, Emma Smith, Emma Belton, Katherine Jeays-Ward, Matt Willox, Nicki Barker, Pete Metherall, Avril McCarthy, Heath Read, Heather Elphick

**Affiliations:** 1 Community Pharmacy, Centre for Primary Care and Public Health (Unisanté), University of Lausanne, Rue du Bugnon 44, 1011 Lausanne, Switzerland; 2 School of Pharmaceutical Sciences, University of Geneva, Rue Michel-Servet 1, 1211 Geneva 4, Switzerland; 3 Institute of Pharmaceutical Sciences of Western Switzerland, University of Geneva, University of Lausanne, Rue Michel-Servet 1, 1211 Geneva 4, Switzerland; 4 Faculty of Health Sciences, Curtin University, Bentley, WA 6102 Australia; 5 Cancer Research Division, Cancer Council NSW, Woolloomooloo, NSW Australia; 6 Faculty of Medicine and Health, The University of Sydney, Camperdown, NSW Australia; 7 Wolfson Palliative Care Research Centre, Hull York Medical School, Faculty of Health Sciences, University of Hull, Hull, HU6 7RX UK; 8 College of Medicine and Health, University of Exeter, Exeter, EX1 2LU UK; 9 Wellcome Centre for Cultures and Environments of Health, University of Exeter, Exeter, EX1 2LU UK; 10 Nuffield Department of Primary Care Health Sciences, University of Oxford, Oxford, OX2 6GG UK; 11 Exeter HS&DR Evidence Synthesis Centre, Institute of Health Research, College of Medicine and Health, University of Exeter, Exeter, EX1 2LU UK; 12 Department of Nutrition & Dietetics, The Prince Charles Hospital, Brisbane, Queensland Australia; 13 School of Human Movement & Nutrition Sciences, The University of Queensland, Brisbane, Queensland Australia; 14 University of Global Health Equity, Kigali, Rwanda; 15 Department of Medical Social Sciences, Feinberg School of Medicine, Northwestern University, Chicago, IL USA; 16 Centre for Implementation Science, Department of Health Services and Population Research, Institute of Psychiatry, Psychology & Neuroscience, Kings College London, London, UK; 17 Centre for Global Mental Health, Department of Health Services and Population Research, Institute of Psychiatry, Psychology & Neuroscience, King’s College London, London, UK; 18 Department of Psychiatry, WHO Collaborating Centre for Mental Health Research and Capacity-Building, School of Medicine, College of Health Sciences, Addis Ababa University, Addis Ababa, Ethiopia; 19 Centre for Innovative Drug Development and Therapeutic Trials for Africa (CDT-Africa), College of Health Sciences, Addis Ababa University, Addis Ababa, Ethiopia; 20 St George’s University of London and Kingston University, Centre for Health and Social Care Research, London, UK; 21 Musculoskeletal Programme, Health Innovation Network, London, UK; 22 Bone & Joint Research Office, Royal Cornwall Hospitals NHS Trust, Truro, UK; 23 St George’s University of London and Kingston University, Faculty of Health, Social Care and Education, London, UK; 24 Health Innovation Network, Musculoskeletal Programme, London, UK; 25 Faculty of Health, Social Care and Education, St George’s University of London and Kingston University, 6th Floor, Hunter Wing, Faculty of Health, Social Care and Education, London, UK; 26 Centre for Implementation Science, Department of Health Services and Population Research, Institute of Psychiatry, Psychology & Neuroscience, Kings College London, London, UK; 27 Centre for Global Mental Health, Department of Health Services and Population Research, Institute of Psychiatry, Psychology & Neuroscience, King’s College London, London, UK; 28 Department of Psychiatry, WHO Collaborating Centre for Mental Health Research and Capacity-Building, School of Medicine, College of Health Sciences, Addis Ababa University, Addis Ababa, Ethiopia; 29 Centre for Innovative Drug Development and Therapeutic Trials for Africa (CDT-Africa), College of Health Sciences, Addis Ababa University, Addis Ababa, Ethiopia; 30 UCL Institute for Global Health, University College London, London, UK; 31 School of Health Sciences, University of East Anglia, Norwich, UK; 32 Kings Global Health Institute, Kings College London, London, UK; 33 Clinical Psychology in Healthcare, Department of Women’s and Children’s Health, Uppsala University, Uppsala, Sweden; 34 Centre for Gender Research, Uppsala University, Uppsala, Sweden; 35 Department of Health Psychology, University Medical Center Groningen, University of Groningen, Groningen, the Netherlands; 36 Department of Psychology, Health and Technology, University of Twente, Enschede, the Netherlands; 37 Division of Community Paediatrics, Department of Paediatrics and Child Health, Faculty of Health Sciences, University of the Witwatersrand, Johannesburg, South Africa; 38 DSI-NRF Centre of Excellence in Human Development, University of the Witwatersrand, Johannesburg, South Africa; 39 Human Sciences Research Council, Durban, South Africa; 40 University College London, MRC Clinical Trials and Methodology Unit, 90 High Holborn, London, WC1V 6LJ England; 41 Medicine Optimisation Research Group, School of Pharmacy and Medical Sciences, University of Bradford, Bradford, BD7 1DP UK; 42 Medicine Management and Pharmacy Services, Leeds Teaching Hospitals NHS Trust, Leeds, LS1 3EX UK; 43 Department of Management, Huddersfield Business School, University of Huddersfield, Huddersfield, HD1 3DH UK; 44 The McPin Foundation, 7-14 Great Dover Street, London, SE1 4YR UK; 45 NIHR MindTech MedTech Co-operative, Institute of Mental Health, School of Medicine, University of Nottingham, Nottingham, UK; 46 NIHR Nottingham Biomedical Research Centre, Mental Health and Technology Theme, University of Nottingham, Nottingham, UK; 47 Centre for Implementation Science, Health Service and Population Research Department, King’s College London, London, UK; 48 Diabetes Department, Guy’s and St Thomas’ NHS Foundation Trust, London, UK; 49 Diabetes Department, Royal Bournemouth and Christchurch Hospitals NHS Foundation Trust, Dorset, UK; 50 Diabetes Department, Sheffield Teaching Hospitals NHS Foundation Trust, Sheffield, UK; 51 Diabetes Department, King’s College Hospital NHS Foundation Trust, London, UK; 52 Centre for Implementation Science, Health Service and Population Research Department, King’s College London, London, UK; 53 Diabetes Department, Guy’s and St Thomas’ NHS Foundation Trust, London, UK; 54 Diabetes Department, Royal Bournemouth and Christchurch Hospitals NHS Foundation Trust, Dorset, UK; 55 Diabetes Department, Sheffield Teaching Hospitals NHS Foundation Trust, Sheffield, UK; 56 Diabetes Department, King’s College Hospital NHS Foundation Trust, London, UK; 57 Center for Implementation Science, Health Service & Population Research Department, Institute of Psychiatry, Psychology & Neuroscience (IoPPN), King’s College London, London, UK; 58 School of Health Sciences, University of East Anglia, Norwich, UK; 59 Department of Urology, Bart’s NHS Trust, Whipps Cross Hospital, London, UK; 60 Australian Institute of Health Innovation, Macquarie University, Sydney, NSW Australia; 61 Murdoch Children’s Research Institute, Melbourne, VIC Australia; 62 Centre for Implementation Science, Health Services and Population Research Department, King’s College London, London, UK; 63 Faculty of Life Sciences and Medicine, King’s College London, London, UK; 64 Faculty of Medicine, Department of Infectious Disease, Imperial College London, London, UK; 65 AMRITA Hospital, Kerala, India; 66 Centre for Implementation Science, Health Services and Population Research Department, King’s College London, London, UK; 67 Faculty of Life Sciences and Medicine, King’s College London, London, UK; 68 Faculty of Medicine, Department of Infectious Disease, Imperial College London, London, UK; 69 AMRITA Hospital, Kerala, India; 70 Division of Infectious Diseases & HIV Medicine at Groote Schuur Hospital, University of Cape Town (UCT), Cape Town, South Africa; 71 Research Evidence into Policy, Programme and Practice (REPPP), School of Law, University of Limerick, Limerick, Ireland; 72 The Center for Implementation, Toronto, Ontario Canada; 73 The Center for Implementation, Toronto, Ontario Canada; 74 King’s College London, London, SE1 7EH UK; 75 Welbodi Partnership, Freetown, Sierra Leone; 76 Ministry of Health and Sanitation, Freetown, Sierra Leone; 77 Department of Collective Health, University of Campinas, Campinas, SP Brazil; 78 Department of Biostatistics and Health Informatics & Health Services and Population Research Department, Centre for Implementation Science, Institute of Psychiatry, Psychology and Neuroscience, Kings College London, London, UK; 79 Department of Nursing and Midwifery, University of Limerick, Limerick, Ireland; 80 Milford Care Centre, Limerick, Ireland; 81 All-Ireland Institute of Hospice and Palliative Care, Dublin, Ireland; 82 Department of Psychiatry and Behavioral Sciences, Northwestern University, Chicago, IL USA; 83 Center for Prevention Implementation Methodology, Northwestern University, Chicago, IL USA; 84 Third Coast Center for AIDS Research, Chicago, IL USA; 85 Institute for Sexual and Gender Minority Health and Wellbeing, Northwestern University, Chicago, IL USA; 86 Department of Medical Social Sciences, Northwestern University, Chicago, IL USA; 87 Department of Preventive Medicine, Northwestern University, Chicago, IL USA; 88 Women’s College Hospital, Toronto, Ontario M5S 1B2 Canada; 89 Ottawa Hospital Research Institute, Ottawa, Ontario K1Y 4E9 Canada; 90 Northwestern University Feinberg School of Medicine, Department of Psychiatry and Behavioral Sciences, Chicago, IL 60611 USA; 91 Northwestern University Feinberg School of Medicine, Medical Social Sciences, Chicago, IL 60611 USA; 92 Department of Epidemiology and Public Health, UCL Institute of Epidemiology and Health Care (IEHC), London, UK; 93 Aceso Global Health Consultants Ltd, London, UK; 94 Population Policy and Practice (PPP), UCL Great Ormond Street Institute of Child Health (GOS-ICH), London, UK; 95 Department of Behavioural Sciences & Health, UCL IEHC, London, UK; 96 UCL Institute for Global Health, London, UK; 97 Tower Hamlets GP Care Group, Mile End Hospital, London, UK; 98 Children’s Health 0-19 Service, Newham Council, London, UK; 99 Whittington Health NHS Trust, London, UK; 100 School of Sport, Exercise & Rehabilitation Sciences, University of Birmingham, Birmingham, UK; 101 Health Sciences, University of York, York, UK; 102 University of Exeter Medical School, Exeter, UK; 103 Royal Cornwall Hospitals NHS Trust, Cornwall, UK; 104 MRC/CSO Social and Public Health Sciences Unit & Robertson Centre for Biostatistics, Institute of Health and Well Being, University of Glasgow, Glasgow, UK; 105 Centre for Global Health and Health Partnerships, King’s College London, London, SE5 9RJ UK; 106 Great Ormond Street Hospital for Children, London, WC1N 3JH UK; 107 Mercy Ships UK, The Lighthouse, Stevenage, SG1 2EF UK; 108 Centre for Implementation Science, Health Service and Population Research Department, Institute of Psychiatry, Psychology and Neuroscience, King’s College London, London, SE5 8AB UK; 109 King’s Health Economics, Health Service and Population Research Department, Institute of Psychiatry, Psychology and Neuroscience, King’s College London, London, SE5 8AB UK; 110 School of Clinical Medicine, University of Cambridge, Cambridge, UK; 111 Academic Neurosurgery Unit, Department of Clinical Neurosurgery, University of Cambridge, Cambridge, UK; 112 Population Health Sciences Institute, Newcastle University, Newcastle upon Tyne, NE2 4AX UK; 113 College of Medicine and Health, University of Exeter, Exeter, EX1 2LU UK; 114 Department of Nursing, Midwifery & Health, Northumbria University, Newcastle upon Tyne, NE7 7XA UK; 115 Department of Health Science and the Amsterdam Public health Institute, Vrije Universiteit, Amsterdam, The Netherlands; 116 Department of Clinical Pharmacology and Pharmacy, Amsterdam UMC, loc. VUmc, Amsterdam, The Netherlands; 117 Netherlands institute for health services research (NIVEL), Utrecht, The Netherlands; 118 Faculty of Science and Engineering, University of Groningen, Groningen, The Netherlands; 119 King’s Centre for Global Health & Health Partnerships, School of Population Health & Environmental Sciences, Faculty of Life Sciences and Medicine, King’s College London, London, UK; 120 Centre for Implementation Science, Health Service and Population Research Department, Kings College London, London, UK; 121 Department of Surgery, College of Medicine and Allied Health Sciences, University of Sierra Leone, Freetown, Sierra Leone; 122 Faculty of Nursing, College of Medicine and Allied Health Sciences, University of Sierra Leone, Freetown, Sierra Leone; 123 Connaught Teaching Hospital Complex, Sierra Leone, Freetown, Sierra Leone; 124 Institute of Applied Health Research, University of Birmingham, Birmingham, UK; 125 Women’s College Hospital Institute for Health System Solutions and Virtual Care, Womens College Hospital, Toronto, Ontario Canada; 126 SickKids Research Institute, Toronto, Ontario Canada; 127 The Peter Gilgan Centre for Women’s Cancers, Womens College Hospital, Toronto, Ontario Canada; 128 Division of Hematology/Oncology, Hospital for Sick Children, Toronto, Ontario Canada; 129 Institute of Health Policy, Management and Evaluation, University of Toronto, Toronto, Ontario Canada; 130 Department of Family and Community Medicine, University of Toronto, Toronto, Ontario Canada; 131 School of Public Health, University College Cork, Western Rd, Cork, Ireland; 132 School of Health Sciences, University of East Anglia, Norwich, UK; 133 Institute for Public Health, Washington University in St. Louis, St. Louis, Missouri USA; 134 Department of Primary Care and Population Health, University College London, London, UK; 135 The National Institute on Minority Health and Health Disparities, The National Institutes of Health, Bethesda, Maryland USA; 136 Department of Prevention and Community Health, George Washington University, Washington, DC USA; 137 West Suffolk NHS Foundation Trust, Bury St. Edmunds, Suffolk, UK; 138 Truth Initiative, Washington, DC USA; 139 Centre for Quality Improvement and Clinical Audit, Royal College of Obstetricians and Gynaecologists, London, SE1 1SZ UK; 140 Royal College of Midwives, London, W1G 9NH UK; 141 Department of Health Services Research and Policy, London School of Hygiene and Tropical Medicine, London, WC1H 9SH UK; 142 Centre for Implementation Science, Health Service and Population Research Department, King’s College London, London, SE5 8AF UK; 143 Croydon University Hospitals NHS Trust, Croydon, CR7 7YE UK; 144 King’s College London, London, Strand WC2R 2LS UK; 145 School of Allied Health, University of Limerick, Limerick, Ireland; 146 Health Implementation Science and Technology Cluster, Health Research Institute, (HIST_HRI) University of Limerick, Limerick, Ireland; 147 National Society for the Prevention of Cruelty to Children, London, UK; 148 Devices for Dignity Med Tech Co-operative, Sheffield Teaching Hospitals NHS Foundation Trust, Sheffield, UK; 149 ACES, Sheffield Hallam University, Sheffield, UK; 150 Sheffield Children’s NHS Foundation Trust, Sheffield, UK; 151 3-D Lab, Sheffield Teaching Hospitals NHS Foundation Trust, Sheffield, UK; 152 Clinical Engineering, Sheffield Teaching Hospitals NHS Foundation Trust, Sheffield, UK

## Institute of Psychiatry, Psychology and Neuroscience, King’s College London, 16^th^ and 17^th^ July 2020

### O1. Implementation study of an inter-professional support programme for patients with type 2 diabetes in a Swiss primary care setting

#### Noura Bawab^1,2,3^, Joanna C. Moullin^4^, Olivier Bugnon^1,2,3, α^, Clémence Perraudin^1, α^

##### ^1^Community Pharmacy, Centre for Primary Care and Public Health (Unisanté), University of Lausanne, Rue du Bugnon 44, 1011 Lausanne, Switzerland; ^2^School of Pharmaceutical Sciences, University of Geneva, Rue Michel-Servet 1, 1211 Geneva 4, Switzerland; ^3^Institute of Pharmaceutical Sciences of Western Switzerland, University of Geneva, University of Lausanne, Rue Michel-Servet 1, 1211 Geneva 4, Switzerland; ^4^Faculty of Health Sciences, Curtin University, Bentley WA 6102, Australia

###### **Correspondence:** Noura Bawab (Noura.bawab@unisante.ch)

^α^ equal co-senior authors

**Background:**

The Swiss federal government promoted the evaluation of an inter-professional patient support model, including regular motivational interviews (patient-pharmacist), medication adherence and patient-reported outcomes monitoring and interactions with physicians. The aim of this 15-month study was to evaluate the implementation process of a programme tailored to patients with type 2 diabetes, taking at least one oral antidiabetic treatment.

**Materials and methods:**

This is a prospective, multi-centric, observational, cohort study using a hybrid implementation-effectiveness design and the Framework for the Implementation of Services in Pharmacy (FISpH) [1]. Outcomes were assessed at each stage of the implementation process using both quantitative and qualitative methods. A set of implementation measures reported on the process (number of pharmacies going through the stages), outcomes (e.g. reach, fidelity) and impact (influencing factors and implementation strategies).

**Results:**

Describes the indicators of progress along the implementation process.

Two-hundred-twelve patients were included to benefit from the support programme in 27 pharmacies. The mean inclusion rate per pharmacy was 8 patients (SD 6, range: 1-29). We observed a step-by-step implementation process: 1) internal organisation: teaching and coaching of the pharmacy team, identification of eligible patients, 2) preparation of inter-professional collaboration: information and local networking with physicians; and 3) relationship building with patients. Main influencing factors were pharmacists’ skills in motivational interviewing, support from pharmacy owners, pre-existing local inter-professional networks and profitability of the programme.

**Conclusions:**

This evaluation provided evidence regarding the implementation capacity and acceptability of the programme by pharmacy teams, patients with diabetes and physicians: a promising start for inter-professional chronic care services.

**Acknowledgements**

We are very grateful to the stakeholders (FOPH, pharmaSuisse, santésuisse, curafutura) for funding the study. We would like to sincerely thank all the pharmacies, patients and physicians for their participation, as well as Aurélien Georges for helping with the facilitation of the focus groups.

**Trial Registration:**

Not applicable

**Consent to publish**

Not applicable.

**Reference**

1. Moullin JC, Sabater-Hernandez D, Benrimoj SI. Model for the evaluation of implementation programs and professional pharmacy services. Res Social Adm Pharm. 2016;12(3):515-22.

**Fig. 1 (abstract O1).**
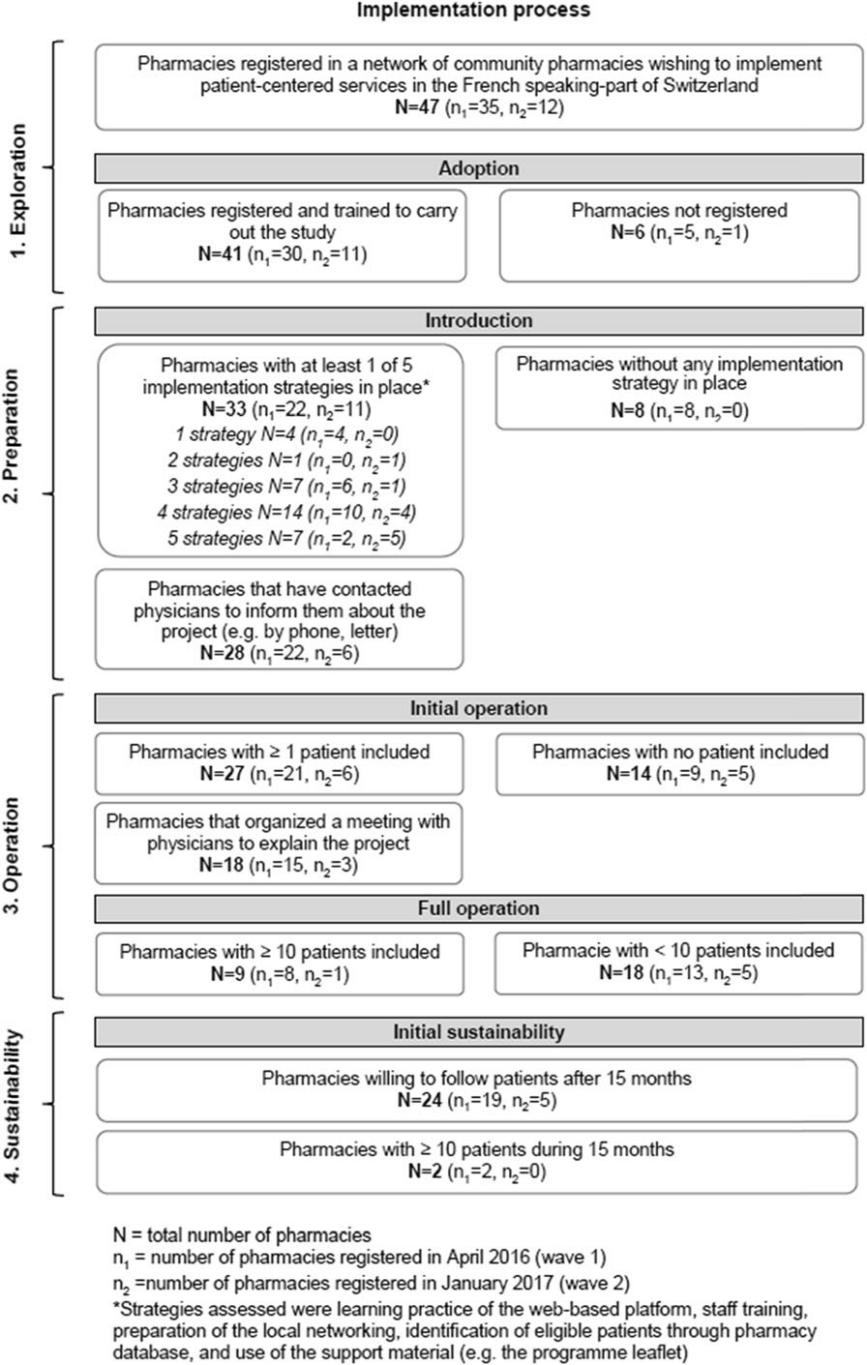
Indicators of progress of the implementation process

### P2. Building capacity from within - upskilling healthcare professionals to lead an evidence-based implementation approach

#### April Morrow^1,2^, Priscilla Chan^1^, Emily Hogden^1^ & Natalie Taylor^1,2^

##### ^1^Cancer Research Division, Cancer Council NSW, Woolloomooloo, NSW, Australia; ^2^ Faculty of Medicine and Health, The University of Sydney, Camperdown, NSW, Australia

###### **Correspondence:** April Morrow (April.morrow@nswcc.org.au)

**Background:**

Translating evidence into complex health systems is an ongoing challenge. Hospital-based implementation trials are often led by external researchers who, despite expertise in implementation science, lack the necessary understanding of the inner workings of the healthcare system. Partnerships with behavioural scientists to upskill healthcare professionals to lead evidence-based implementation approaches may be more sustainable for effective translation. However, limited guidance exists on the implementation training and support needs of healthcare professionals, and few training programs have been described or evaluated to date.

**Method:**

The *Hide and Seek Project* (*HaSP*) is a cluster randomised controlled trial testing two implementation approaches for improving hereditary cancer referral at eight Australian hospitals. Nine healthcare professionals were recruited from hospitals as ‘Implementation Leads’ and trained via a one-day workshop. Ongoing support is provided by the researchers via teleconferencing prior to key study activities. Implementation Leads participated in semi-structured interviews to explore their perceptions of the training program. Interview transcripts were analysed using inductive thematic analysis. Training contents will be presented.

**Results:**

Nine Implementation Leads from various professional backgrounds completed the workshop, all of whom participated in post-training interviews. Four key themes were identified: (1) workshop feedback, (2) knowledge and skills, (3) implementation barriers and facilitators, and (4) building health system capacity for implementation. The workshop was positively received, and participants felt well-supported by the research team. A number of areas for improvement were identified.

**Conclusion:**

Whilst maximising the potential for *HaSP* trial success, this training has the potential for a prolonged impact within the health system, with healthcare professionals having gained knowledge and skills they felt were transferrable to other clinical contexts. Insights from the evaluation will guide future efforts to deliver training on a larger scale across a range of healthcare contexts.

**Trial Registration:** ACTRN12618001072202

**Consent to publish**

All participants consented for publication

### O3. How can strategies to address mental ill-health in doctors and medical students be optimised? The interplay of intervention and implementation identified in the ‘Care Under Pressure’ realist review

#### Mark Pearson^1^, Daniele Carrieri^2,3^, Karen Mattick^2^, Chrysanthi Papoutsi^4^, Simon Briscoe^5^, Geoff Wong^4^, Mark Jackson^2^

##### ^1^Wolfson Palliative Care Research Centre, Hull York Medical School, Faculty of Health Sciences, University of Hull, Hull, HU6 7RX, UK; ^2^College of Medicine and Health, University of Exeter, Exeter, EX1 2LU, UK; ^3^Wellcome Centre for Cultures and Environments of Health, University of Exeter, Exeter, EX1 2LU, UK; ^4^Nuffield Department of Primary Care Health Sciences, University of Oxford, Oxford, OX2 6GG, UK; ^5^Exeter HS&DR Evidence Synthesis Centre, Institute of Health Research, College of Medicine and Health, University of Exeter, Exeter, EX1 2LU, UK

###### **Correspondence:** Mark Pearson (mark.pearson@hyms.ac.uk)

**Background:**

The impact of the work environment on the mental health of doctors is internationally recognised. However, research syntheses on interventions that provide support, advice and/or treatment to sick doctors have not fully taken account of intervention complexity and heterogeneity, the multiple dimensions of the issue, nor the challenges of implementing strategies to address mental ill-health in doctors. We: 1) conducted a realist review of interventions to improve doctors’ and medical students’ mental ill-health, engaging throughout with a diverse group of stakeholders; 2) developed recommendations to support tailoring, implementation, monitoring and evaluation of these strategies.

**Method:**

Realist review, conducted and reported consistent with RAMESES standards. Research and policy sources identified through bibliographic database searches, purposive searches, and stakeholder engagement. Extracted data analysed using a realist lens to identify explanatory context-mechanism-outcome configurations (CMOcs) of mental ill-health in doctors and medical students.

**Results:**

179 sources were included, 45% of which were from the USA and 74% of which were published in 2009 or later. The synthesis produced 19 CMOcs (processes, relationality, balance, and implementation) explaining how mental ill-health develops in the workplace and how strategies can be implemented to reduce mental ill-health. Trust was identified as highly important in explaining the interplay between implementation strategy, intervention development, and the broader workplace context.

**Conclusion:**

Interventions to improve doctors’ and medical students’ mental ill-health should take account of the complexity of the issue and its implementation by operating at multiple levels and engaging diverse stakeholders. Refining existing complex interventions, informed by the CMOcs in this review, is likely to be more efficient and ultimately more effective than developing new interventions. This review has demonstrated the importance of Realist review in critically synthesising diverse evidence about complex health service issues so that implementable multi-level strategies can be developed.

**Acknowledgements**

This project is funded by the by the National Institute for Health Research (NIHR) HS&DR (project number 16/53/12) and supported by the NIHR Collaboration for Leadership in Applied Health Research and Care South West Peninsula. The views expressed are those of the author(s) and not necessarily those of the NHS, the NIHR or the Department of Health and Social Care.

**Systematic review registration:** PROSPERO CRD42017069870

### O4. Identifying embedded, low-value nutrition care practices for de-implementation - a nominal group technique approach

#### Alita Rushton^1^, Kai Elmas^1^, Dr Jack Bell^1,2^

##### ^1^Department of Nutrition & Dietetics, The Prince Charles Hospital, Brisbane, Queensland, Australia; ^2^School of Human Movement & Nutrition Sciences, The University of Queensland, Brisbane, Queensland, Australia

###### **Correspondence:** Jack Bell (jack.bell@health.qld.gov.au)

**Background:**

Traditional malnutrition models of care apply individualised, dietitian administered nutrition care. Shifting to systematised and/or interdisciplinary alternatives is an important step towards improved service efficiencies, effectiveness, and patient reported experience measures. This study consequently applied a nominal group technique approach with experts in field to identify and prioritise low-value nutrition care practices for de-implementation.

**Method:**

Workshops using the nominal group technique were undertaken at eight hospitals across Queensland, Australia administered by a single experienced clinician/implementation expert. Purposively sampled dietitians and nutrition assistants were asked the question “What highly individualised malnutrition care activities do you think we could replace with systematised, interdisciplinary malnutrition care?”. Each participant was provided opportunity to individually list and present responses, discuss them as a group, and then vote for the highest priorities; each participant was allowed five votes.

**Results:**

Nine workshops were conducted across eight sites. Dietitians (51) and assistants (12) identified 101 dietetics actions to replace with systematised, interdisciplinary alternatives. These were spread across screening (n=5), assessment (n=31), diagnosis (n=2), intervention (n=45), and monitoring and evaluation (n=18) domains of the nutrition care process. Actions that received the highest number of nominal group technique votes were: comprehensive dietitian assessments for low risk referrals (n=50); dietetics follow-up reviews where unlikely to add substantial benefit (n=32); individualised inpatient educations by dietitian where specialised education or counselling were considered low-value (n=28); individualised food and fluid support for patients who do not require specialised dietitian care (n=22); and assistants undertaking malnutrition screening (n=19).

**Conclusion:**

Findings highlight the nominal group technique as a useful approach to prioritising embedded, low-value clinical care activities for de-implementation. The individual and group elements of the framework supported establishment of consensus amongst practicing clinicians and policy makers. These findings are currently supporting practice and policy value-based healthcare reform across Queensland hospitals.

**Acknowledgements**

This program has received funding support from Allied Health Professions Office, Queensland, and the Australian Government Medical Research Futures Fund

### O5. Bridging the Implementation Gap: Using implementation research to understand how six countries implemented evidence-based interventions to reduce amenable under-5 mortality

#### Agnes Binagwaho^1^, Miriam F Frisch^1^, Jovial Thomas Ntawukuriryayo^1^, Dieudonné Nkurunziza^1^, Kelechi Udoh^1^, Amy VanderZanden^1^, Laura Drown^1^, Lisa R Hirschhorn^2^

##### ^1^University of Global Health Equity, Kigali, Rwanda; ^2^Department of Medical Social Sciences, Feinberg School of Medicine, Northwestern University, Chicago, IL, USA

###### **Correspondence:** Agnes Binagwaho (abinagwaho@ughe.org)

**Background:**

Despite health-system evidence-based interventions (EBIs) known to reduce amenable under-5 childhood mortality (U5M), countries struggle to effectively bridge the implementation gap. We applied implementation research (IR) to understand how six countries (Rwanda, Senegal, Ethiopia, Bangladesh, Nepal, Peru) implemented EBIs to contribute to successfully dropping U5M.

**Method:**

We developed an IR framework building on the Exploration, Preparation, Implementation, Sustainment Framework and Consolidated Framework for Implementation Research to include adaptation (EPIAS) and capture contextual factors at the global, national, health system, and individual levels.[1,2] We used mixed methodology to analyze EBI implementation and successes and challenges.

**Results:**

These countries took a five-step implementation approach, reflecting EPIAS, to implement the EBIs, recognizing contextual factors needing to be addressed or influencing implementation strategies.

1. **Exploration:** Understand and research the problem and possible solutions; identify multisectoral stakeholders; identify possible implementation strategies.
2. **Preparation:** for implementation: Choose EBIs or implementation strategies that fit national contexts, priorities, and identified gaps; identify contextual factors to be addressed or influencing strategies; develop evidence-based guidelines for implementation; plan for monitoring and evaluation.
3. **Initiate implementation:** Disseminate national protocols; train personnel and stakeholders; implement interventions and accountability frameworks; monitor implementation; follow research and new guidelines to identify new EBIs or strategies.
4. **Adaptation:** Use monitoring data and stakeholder engagement to determine gaps in fidelity, reach, acceptability, and effectiveness; make evidence-based adaptations; continue monitoring to assess impact; identify new resources needed.
5. **Sustainment:** Ensure longer-term funding; cultivate a culture of evaluation and implementation of needed changes; integrate training and capacity-building.

These countries’ success in U5M reduction also included broader initiatives to reduce disease burden (ex. sanitation) and improve child and family resilience (ex. economic development, female empowerment).

**Conclusion:**

These findings provide lessons for other countries to better adapt and implement health-system delivered EBIs to accelerate U5M reduction and achieve equity.

### P6. Protocol for participatory, theory-informed design of an implementation research programme for health system strengthening to improve the delivery of maternal, surgical and primary care in sub-Saharan Africa (ASSET)

#### Seward N^1*^, Araya R^2^, Hanlon C^2,3,4^, Harding R^5^, Hinrichs-Krapels S^6,7^, Lund C^2,6^, Mayston R^2^, Murdoch J^8^, Thornicroft G^1,2^, Gao W^5^, Radhakrishnan M^1^, Sevdalis N^1^

##### ^1^Centre for Implementation Science, Health Service and Population Research Department, Institute of Psychiatry, Psychology & Neuroscience, King’s College London, UK; ^2^Centre for Global Mental Health, Health Service and Population Research Department, Institute of Psychiatry, Psychology & Neuroscience, KCL, UK; ^3^Department of Psychiatry, WHO Collaborating Centre for Mental Health Research and Capacity-Building, School of Medicine, College of Health Sciences, Addis Ababa University, Ethiopia; ^4^Centre for Innovative Drug Development and Therapeutic Trials for Africa (CDT-Africa), College of Health Sciences, Addis Ababa University, Ethiopia; ^5^Cicely Saunders Institute Of Palliative Care, Policy & Rehabilitation, KCL, UK; ^6^King’s Global Health Institute, KCL, UK; ^7^The Policy Institute, KCL, UK; ^8^School of Health Sciences, University of East Anglia, UK

###### **Correspondence:** Seward N (nadine.seward@kcl.ac.uk)

**Background:**

Achieving Universal Health Coverage that includes the availability and delivery of high-quality evidence-based care has been identified as a priority for health system strengthening (HSS) in Low- and Middle-Income Countries (LMICs) [1]. ASSET is an implementation research programme for HSS working on three care platforms across four sub-Saharan African countries; Ethiopia, Sierra Leone, South Africa, and Zimbabwe.

The overall aim of the implementation science theme within ASSET is to advance our understanding of how to design and evaluate HSS interventions across different health systems and contexts to: (1) understand what implementation strategies work, for whom and how, and (2) improve implementation science methodologies applied to such HSS interventions.

**Methods:**

Using a mixed-method approach we will use implementation determinant and evaluation frameworks as part of ‘effectiveness-implementation hybrid trial’ designs to evaluate ASSET programme interventions. The *pre-implementation phase* will collect information on contextual barriers and/or enablers that influence selection of different HSS interventions. The *implementation and evaluation phase* will evaluate: (1) effectiveness of implementation strategies (based on standardised implementation outcomes assessment), (2) influence of context on the effectiveness of implementation strategies in delivering the interventions, and (3) influence of context on the mechanisms introduced by the interventions to produce improvement.

To facilitate comparisons between countries/platforms, we will adapt the ‘matrixed multiple case study’ approach [2]. This methodology organises, analyses and presents common and heterogeneous findings across implementation sites, in order to seek generalizable knowledge regarding what and how local factors influence implementation.

**Results:** This is a protocol to design and evaluate HSS interventions, so as such no results are applicable.

**Conclusions:**

This research programme will create a compilation of implementation strategies used in LMIC contexts and compare the associated barriers and the effectiveness on implementation outcomes. Given this is one of the first large scale programmes to design and evaluate HSS interventions across multiple study sites, we hope to use this opportunity to address key methodological challenges associated with such programmes.

**References**

1. Kruk M, Gage A, Arenault C. High quality health systems n the Sustainable Development Goal era: time for a revolution. Lancet Global Health. 2018; 6(11). 10.1016/s2214-109x(18)30386-3

2. Kim, B, Sullivan J, Ritchie M. Comparing variations in implementation process and influences across multiple sites: what works, for whom, and how? Psychiatry Research. 2019; 283. 10.1016/j.psychres.2019.112520

### O7. Delivering NICE Joint Pain Advice in the workplace

#### Mike Hurley^1^, Sally Irwin^2^, Jo Erwin^3^, Fay Sibley^2^, Amber Gibney^2^, Andrea Carter^2^

##### ^1^St George's University of London and Kingston University, Centre for Health and Social Care Research, London, United Kingdom; ^2^Musculoskeletal Programme, Health Innovation Network, London, United Kingdom; ^3^Bone & Joint Research Office, Royal Cornwall Hospitals NHS Trust, Truro, United Kingdom

###### **Correspondence:** Mike Hurley (michael.hurley@sgul.kingston.ac.uk)

**Background:**

NICE recommend people with knee, hip and/or back pain receive self-management and lifestyle advice, emphasising the importance of physical activity and maintaining healthy weight. Unfortunately, delivering NICE advice to the millions of people requiring help is prevented by limitations in time, facilities and expertise. Moreover, using healthcare facilities and professionals medicalises a problem most people see as a natural part of living and ageing. Joint Pain Advice (JPA) can deliver NICE advice in a variety of health and community settings, using a range healthcare and non-healthcare professionals [1,2]. Here we extend JPA delivery into workplace settings using local health champions.

**Method:**

In workplaces, 2-3 people were trained to deliver JPA. This involved an initial assessment of participant’s pain (using VAS), musculoskeletal health and function (MSK-HQ), activity level (number of days/week active for >30mins). Participants were taught simple self-management strategies, encouraged to adopt healthier lifestyle using motivational interviewing, goal-setting action/coping planning and personalised care plans constructed. Participants were reviewed 3 times over 6-months, baseline outcomes reassessed, progress highlighted, health messages reinforced and plans and care plans revised if necessary. Results presented as mean change (95% CI)

**Results:**

Twelve large public organisations or small/medium private enterprises delivered JPA to 417 people. Participants attendance was 75%, suggesting they found JPA acceptable, valued advice tailored to their individual needs and experienced tangible benefits. Overall the MSK-HQ improved by 8 points (CI 6.6 to 9.2), pain 1 (-1.33 to -0.88), activity for >30mins by 1.5 (1.1 to 1.8), self-reported physical function by 1.5 (1.1 to 1.8).

**Conclusion:**

Delivering NICE advice for management of chronic joint pain through JPA in workplace settings using local health champions is practicable, beneficial and valued. This can avoid medicalising the problem and “prevent turning people into patients”. JPA could benefit small medium or large employers across the UK.

**Acknowledgements**

This work was funded by the Department of Work and Pensions Work and Health Unit Challenge Fund

**References**

1. Hurley M V., Semple A, Sibley F, Walker A. Evaluation of a health trainer–led service for people with knee, hip and back pain. Perspect Public Health. 2019;139(6):308–15.

2. Health Innovation Network. Joint Pain Advice – A Model of Care for Chronic Joint Pain [Internet]. [cited 2020 Jun 26]. Available from: https://healthinnovationnetwork.com/projects/joint-pain-advisor-exploring-a-new-model-of-care-for-chronic-joint-pain/

### P8. Increasing access to rehabilitation for knee and hip osteoarthritis – extending ESCAPE-pain delivery to community venues

#### M. Hurley^1, 2^, M. Connelly^2^, H. Sheldon^2^, A. Gibney^2^, R. Hallett^3^, A. Carter^2^

##### ^1^St George’s University of London and Kingston University, Faculty of Health, Social Care and Education, London, United Kingdom; ^2^Health Innovation Network, Musculoskeletal Programme, London, United Kingdom; ^3^Faculty of Health, Social Care and Education, St George’s University of London and Kingston University, 6th Floor, Hunter Wing, Faculty of Health, Social Care and Education, London, United Kingdom

###### **Correspondence:** M. Hurley (michael.hurley@sgul.kingston.ac.uk)

**Background:**

ESCAPE-pain is a rehabilitation programme for knee and/or hip osteoarthritis [1,2]. It is usually delivered in physiotherapy departments, but NHS constraints limits access to the programme. Delivering ESCAPE-pain in community venues could increase accessibility and provide on-going support (3). This study extended delivery of ESCAPE-pain into community venues and evaluated its effectiveness and participant’s experiences.

**Method:**

We trained 369 exercise professionals to deliver ESCAPE-pain in 41 community centres. Pain, function and quality of life (using Knee or Hip Osteoarthritis Outcome Score, K/HOOS) and self-reported activity levels (minutes/week) were measured before and after the programme. Semi-structured interviews estimated people’s experiences of the programme.

**Results:**

386 participants were recruited, mean age 70 years. Before the programme only 24% of participants were “active”, i.e. doing >30 mins activity/week, after participating almost 78% were “active” doing >30 mins/week. Participants reported improvements in pain (10 K/HOOS points; p<0.0001), function (9pts; p<0.0001), QoL (10pts; p<0.0001). These improvements enabled people to walk better, farther, without aids and reduced their social isolation. Better understanding of their problems, ability to self-manage their problems and exercise self-efficacy (the confidence to use exercise to control pain and its impact), made people much more optimistic and they described “the world was a brighter place”. Concomitant with these improvements overall healthcare utilisation reduced. Because they enjoyed the programme most participants were planning to continue exercising to try to maintain these benefits, had joined classes and taken up activities (swimming, golf, walking, yoga).

**Conclusion:**

ESCAPE-pain can be safely delivered by exercise professionals as a community-based rehabilitation programme, it retains its effectiveness and nurtures habitual exercise in participants. As a community-based programme will enable many more people to access the programme and benefit. As a result of this study ESCAPE-pain is now being in many more community venues across the UK [3].

**Acknowledgements**

This work was supported by grants from Sport England, Versus Arthritis and the Health Innovation Network (South London's Academic Health Science Network).

**References**

1. ESCAPE. Enabling Self-management and Coping with Arthritic Pain using Exercise [Internet]. [cited 2020 Jun 20]. Available from: https://escape-pain.org/

2. Hurley M V, Walsh NE, Mitchell H, Nicholas J, Patel A. Long-term outcomes and costs of an integrated rehabilitation program for chronic knee pain: A pragmatic, cluster randomized, controlled trial. Arthitis Care Res. 2012;64(2):238–47.

3. Hurley MV, Carter A. ESCAPE-into the community - A community-based rehabilitation programme for elderly people with chronic joint pain. Perspect Public https://escape-pain.org/Health. 2016;136(2)

### O9. Contextual equipoise: a novel concept to inform ethical implications for implementation research using randomised controlled trials in low- and middle-income countries

#### Seward N^1^, Hanlon C^2,3,4^, Colbourn T^5&^, Murdoch J ^6&^, Prince M^7^, Venkatapuram S^7^, Sevdalis N^1^

##### ^1^Centre for Implementation Science, Department of Health Services and Population Research, Institute of Psychiatry, Psychology & Neuroscience, Kings College, UK; ^2^Centre for Global Mental Health, Department of Health Services and Population Research, Institute of Psychiatry, Psychology & Neuroscience, King’s College London, UK; ^3^Department of Psychiatry, WHO Collaborating Centre for Mental Health Research and Capacity-Building, School of Medicine, College of Health Sciences, Addis Ababa University, Ethiopia; ^4^Centre for Innovative Drug Development and Therapeutic Trials for Africa (CDT-Africa), College of Health Sciences, Addis Ababa University, Ethiopia; ^5^ UCL Institute for Global Health, University College London, UK; ^6^ School of Health Sciences, University of East Anglia, UK; ^7^Kings Global Health Institute, Kings College London, UK

###### **Correspondence:** Seward N (nadine.seward@kcl.ac.uk)

^&^Equal contributors

**Background:**

The call for universal health coverage within low-and middle-income countries, requires the implementation and scale-up of interventions that are known to be effective.^1^ Achieving universal health coverage requires robust implementation research (IR) that evaluates the influence of context on the effectiveness of interventions to deliver evidence-based care [1]. However, where IR uses a randomised controlled trial (RCT) to test the effectiveness of interventions to deliver care that is known to be effective, clinical equipoise may no longer be relevant [2].

IR is fundamentally about evaluating the influence of context on the effectiveness of interventions to deliver evidence-based care [3]. However, the process of conceptualising whether there is sufficient evidence about context to generalise findings from previous research to a new setting is rarely reported, leaving uncertainty as to whether an RCT is justified. This raises important ethical concerns surrounding participants in the control arm of an RCT being exposed to unnecessary harms associated with denying individuals access to care that is known or can be expected to be effective, in the local context [2].

**Proposed methods to address ethical concerns:**

To address this ethical concern, we propose a complementary approach to clinical equipoise for IR, known as *“contextual equipoise.”* We further propose that IR that uses an RCT needs to clearly articulate the grounds for contextual equipoise. However, the process of understanding contextual equipoise raises ontological and epistemological challenges for assessing the certainty of evidence. We discuss these challenges and argue that a guiding principle should be uncertainty amongst key stakeholders, as to the influence of context on the delivery evidence-based care.

**Results:**

This abstract proposes a complementary approach to clinical equipoise for IR, so it is in preliminary stages.

**Conclusions:**

To guide researchers, we describe how theory-driven methods can be applied to help understand if contextual equipoise is justified. We hope our approach helps researchers to better understand and ensure the ethical principle of beneficence is upheld in the real-world contexts of IR in low-resource settings.

**Acknowledgements**

We would like to thank Shalini Ahuja and Leila Younes for reviewing the manuscript and providing insight as to the salience of this topic for implementation researchers in Global Health.

**References**

1. Peterson HB, Haidar J, Fixsen D, Ramaswamy R, Weiner BJ, Leatherman S. Implementing Innovations in Global Women's, Children's, and Adolescents’ Health: Realizing the Potential for Implementation Science. Obstetrics & Gynecology. 2018;131(3):423-30.

2. World Health Organization. Ethics in implementation researcy. Facilitator’s guide. Geneva: World Health Organization; 2019. https://apps.who.int/iris/bitstream/handle/10665/325608/9789241515375-eng.pdf?ua=1 (accessed Dec 2019).

3. Peters DH, Adam T, Alonge O, Agyepong I, Tran N. Implementation research: what it is and how to do it. BMJ : British Medical Journal. 2013;347:f6753.

### P10. Implementation of e-mental health interventions for informal caregivers of adults with chronic diseases: a systematic review

#### Chelsea Coumoundouros^1^, Erika Mårtensson^1,2^, Giulia Ferraris^3^, Louise von Essen^1^, Robbert Sanderman^3,4^, Joanne Woodford^1^

##### ^1^Clinical Psychology in Healthcare, Department of Women’s and Children’s Health, Uppsala University, Uppsala, Sweden; ^2^Centre for Gender Research, Uppsala University, Uppsala, Sweden; ^3^Department of Health Psychology, University Medical Center Groningen, University of Groningen, Groningen, the Netherlands; ^4^Department of Psychology, Health and Technology, University of Twente, Enschede, the Netherlands

###### **Correspondence:** Chelsea Coumoundouros (chelsea.coumoundouros@kbh.uu.se)

**Background:**

Many informal caregivers experience mental health difficulties. E-mental health interventions offer effective and accessible mental health support; however, these interventions are often not implemented. To explore implementation of e-mental health interventions for informal caregivers, a systematic review was conducted to (1) examine implementation barriers and facilitators, and (2) identify implementation and intervention features associated with intervention effectiveness.

**Method:**

Multiple electronic databases were searched for studies published since 2007 reporting on the implementation and/or effectiveness of e-mental health interventions for informal caregivers of adults with chronic diseases. A thematic synthesis of data related to implementation will be used to identify implementation barriers and facilitators. A qualitative comparative analysis, using data from pragmatic randomized controlled trials, will be used to determine combinations of conditions related to an intervention’s implementation or program features, sufficient for intervention effectiveness.

**Results:**

Electronic database searches yielded 9248 unique records to undergo title/abstract screening. The literature screening process is currently underway to identify full-texts eligible for inclusion in the analysis. Preliminary findings will be presented. Implementation barriers and facilitators identified in the thematic synthesis will be presented. These barriers and facilitators will be linked to initial results from the qualitative comparative analysis, as barriers and facilitators may relate to conditions important for intervention effectiveness. Practical applications of these findings will be discussed. If a qualitative comparative analysis cannot be completed prior to the conference, pragmatic trials reporting on intervention effectiveness will be descriptively summarized and analysis plans discussed.

**Conclusions:**

This review will identify key factors to consider during implementation of e-mental health interventions for informal caregivers and present potential solutions to overcome implementation barriers. These findings can be used to inform intervention design and implementation strategies to facilitate the implementation of e-mental health services for informal caregivers.

**Acknowledgements**

This work was supported by the European Union’s Horizon 2020 research and innovation program under the Marie-Sklodowska Curie grant agreement No 814072.

### P11. The importance of stakeholder engagement in the development and implementation of novel interventions in lower-resourced settings: Experiences from the Healthy Pregnancy, Healthy Baby (HPHB) Study

#### Slemming W^1^, Drysdale R^2^, Makusha T^3^ & Richter L^2^

##### ^1^Division of Community Paediatrics, Department of Paediatrics and Child Health, Faculty of Health Sciences, University of the Witwatersrand, Johannesburg, South Africa; ^2^DSI-NRF Centre of Excellence in Human Development, University of the Witwatersrand, Johannesburg, South Africa; ^3^Human Sciences Research Council, Durban, South Africa

###### **Correspondence:** Slemming W (Wiedaad.Slemming@wits.ac.za)

**Background:**

The 2015 South African Department of Health and the 2016 World Health Organization’s antenatal care guidelines include the recommendation of a routine pregnancy ultrasound before 24 weeks.

The HPHB study in Soweto, South Africa, uses scientific evidence on the value of early ultrasounds as a basis for designing an intervention that capitalises on the socio-emotional responses of prospective parents to images of their foetus’s development, the sound of their heartbeat and images that they can share with family and friends. The intervention is embedded in routine health services at Chris Hani Baragwanath Hospital (CHBH) and is being tested through a randomised controlled trial with evaluation of benefits for parents and children at 6 weeks and 6 months’ follow-up. This ongoing study employed multilevel stakeholder engagement strategies during early conceptualisation and development, as well as throughout implementation of the trial.

**Method:**

Stakeholder engagement included meetings and presentations with health policy and management at national and provincial levels; management, clinicians and clerical staff at the referring hospital; district and ward health service staff, non-governmental organizations , academics and researchers.

**Results:**

Formative research conducted with pregnant women attending antenatal clinic at CHBH was key to the intervention development and design. Close collaborations were established with the clinical services at CHBH to ensure efficient and effective recruitment practices and clinical oversight of the trial procedures. Ongoing consultation with a key stakeholder network comprising policy makers, programme implementers, academics, researchers and representatives of multilateral and public benefit organisations, inform intervention procedures and strategies to address challenges that arise during trial implementation. Implementation is monitored and informed through ongoing reflection from staff and formal and informal feedback received from participants.

**Conclusion:**

Meaningful and effective stakeholder engagement is necessary for the development and translation of promising interventions that can be integrated into routine health services, especially in lower-resourced settings.

**Acknowledgements**

The authors would like to thank Chris Hani Baragwanath Hospital and the MRC-Wits Developmental Pathways for Health Research Unit (DPHRU) for facilitating the study

**Trial Registration:** The trial is registered through the Pan African Clinical Trials Registry (PACTR201808107241133).

### O12. An emergency response to the covid-19 pandemic: the Cyprus situation

#### Pallari Elena (elena.pallari@outlook.com)

##### University College London, MRC Clinical Trials and Methodology Unit, 90 High Holborn, London WC1V 6LJ, England

**Background:**

The translation of scientific findings in healthcare is notoriously slow, except for a few ground-breaking innovations, healthcare interventions and recently the global response to the public health emergency posed by the novel coronavirus (covid-19).

**Materials and methods:**

In this study, I evaluated the government plans of the Republic of Cyprus in response to the covid-19 outbreak. I applied the Promoting Action on Research Implementation in Health Services (PARiHS) framework to guide our assessment, using the data provided by the Department of Medical and Public Health Services of the Ministry of Health, the Press and Information Office and publicly available data to assess the healthcare capacity to the pandemic response.

**Results:**

The initiative of the government of the Republic of Cyprus has been responsive and reactive but not proactive, following the robust and vigorous prototype set by the Chinese government. As soon as the first two covid-19-positive cases were diagnosed on the island, the appropriate services were activated, and a thorough contact-tracing lead to the collection of 195 samples (Figure 1). This was the turning point for the government to initiate immediate measures to slow the spread within the community.

**Conclusions:**

As the hub of covid-19 shifted from China to Italy, with Europe becoming the epicentre of the disease, so did the evidence and sharing of best practices in dealing with the pandemic. The PARiHS framework was a useful model to map the spontaneous practice-based implementation plans of the Cypriot government to protect the health of an island with a population of under a million residents.

**Keywords:**

covid-19, Cyprus, healthcare capacity, PARiHS framework

**Acknowledgements**

The author would like to thank Dr Christina Yiannaki, Permanent Secretary at the Department of Medical and Public Health Services of the Ministry of Health.

**Fig. 2 (abstract O12).**
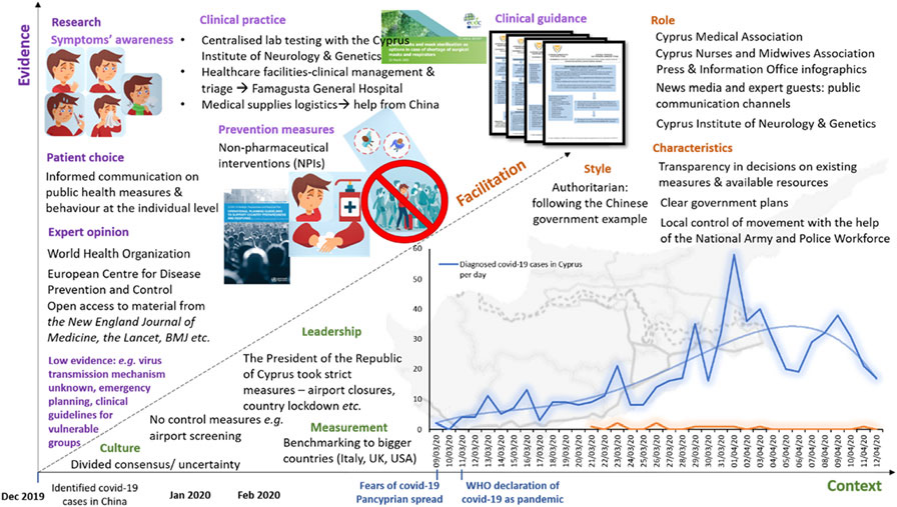
The analysis of the evidence, facilitation and context of the response to the covid-19 in Cyprus using the Promoting Action on Research Implementation in Health Services (PARiHS) framework

### P13. A systematic review of barriers and enablers to the uptake of new medicines

#### Kristina Medlinskiene^1,2^, Justine Tomlinson^1,2^, Iuri Marques^1^, Susan Richardson^3^, Katherine Striling^2^, and Duncan Petty^1^

##### ^1^Medicine Optimisation Research Group, School of Pharmacy and Medical Sciences, University of Bradford, Bradford, United Kingdom, BD7 1DP, UK; ^2^Medicine Management and Pharmacy Services, Leeds Teaching Hospitals NHS Trust, Leeds, United Kingdom, LS1 3EX, UK; ^3^Department of Management, Huddersfield Business School, University of Huddersfield, Huddersfield, HD1 3DH, UK

###### **Correspondence:** Kristina Medlinskiene (k.medlinskiene1@bradford.ac.uk)

**Background:**

Implementation of innovations in healthcare, including new medicines, in the United Kingdom is often lacking behind other countries [1]. The slow uptake of new medicines can delay improvements in patient care and healthcare efficiency. This systematic review aimed to identify factors affecting the uptake of new medicines into practice within healthcare organisations.

**Method:**

The systematic review followed the developed protocol registered with PROSPERO database (CRD42018108536).

**Results:**

The search yielded 35,806 unique titles. Screening of titles and abstracts resulted in 151 papers for full-text review, which further excluded 113 papers. Eleven studies were identified after screening references and citations of included studies. A total of 49 studies were included in the review. The majority of the studies (n=47) were quantitative. Most of the studies (n=36) used secondary data from various databases, e.g. insurance databases. The methodological quality of studies ranged from 45% to 81% with a mean score of 67%. The review findings were grouped into five thematic areas: patient, prescriber, drug, organisational, and external environment factors (Figure 1). Of the five thematic areas coded, organisational, external environment, prescriber and patient factors were the most frequently discussed in the reviewed studies.

**Conclusions:**

The systematic review highlighted various factors affecting the uptake of new medicines. However, factors related to behaviour change were scarcely studied in the reviewed studies. Our further research builds on and explores the review findings using a qualitative approach to identify factors that may not be present in the secondary data, for instance factors related to behaviour change.

**Acknowledgements**

This research work was supported by funding from Leeds Teaching Hospitals NHS Trust and Pharmacy Research UK.

**Reference**

1. Office for Life Sciences and Welcome Trust. Accelerated Access Review: Final Report [Internet]. 2016 [cited 10^th^ March 2019]. Available from: https://assets.publishing.service.gov.uk/government/uploads/system/uploads/attachment_data/file/565072/AAR_final.pdf

**Fig. 3 (abstract P13).**
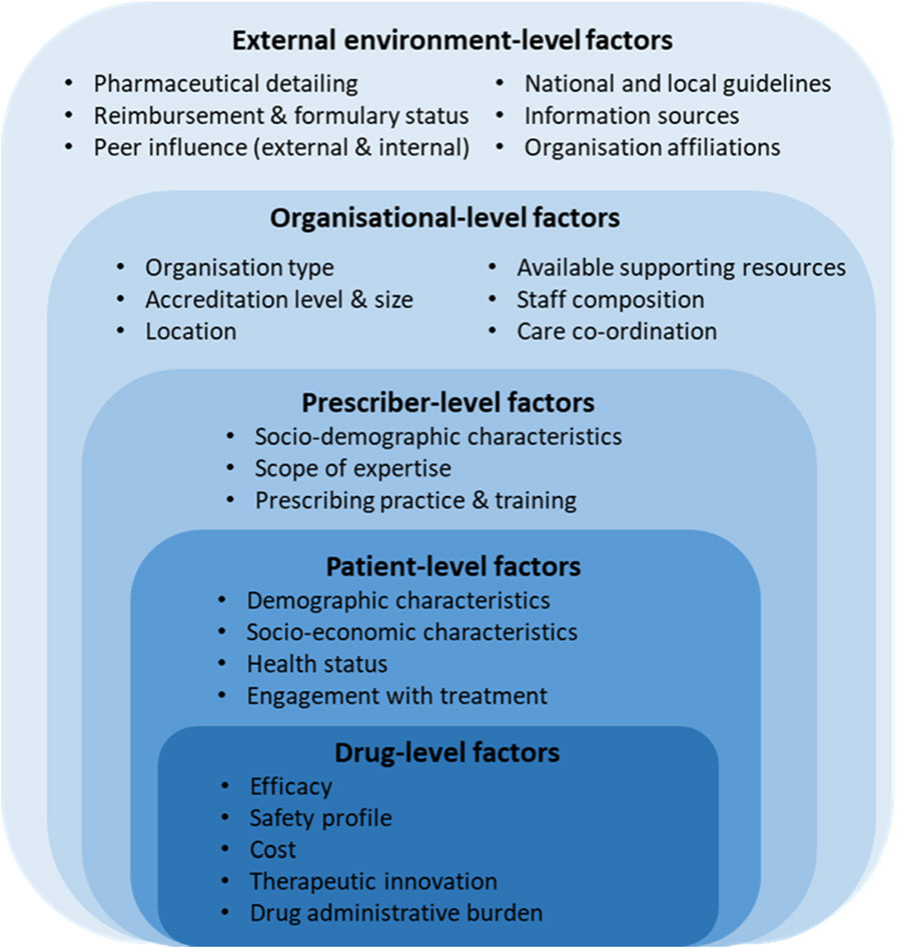
Summary of identified factors in reviewed studies affecting the uptake of new medicines

### P14. Relevant involvement and how it can improve implementation for novel digital mental health interventions

#### Humma Andleeb^1^, Aislinn Bergin^2, 3^, Dan Robotham^1^, Sue Brown^2, 3^, Jennifer Martin^2, 3^

##### ^1^ The McPin Foundation, 7-14 Great Dover Street, London, SE1 4YR, United Kingdom**;**^2^ NIHR MindTech MedTech Co-operative, Institute of Mental Health, School of Medicine, University of Nottingham, Nottingham, United Kingdom**;**^3^ NIHR Nottingham Biomedical Research Centre, Mental Health and Technology Theme, University of Nottingham, Nottingham, United Kingdom

###### **Correspondence:** Humma Andleeb (hummaandleeb@mcpin.org)

**Background:**

gameChange is the NIHR 2017 Mental Health Challenge Award winning project, exploring whether a virtual reality therapy (VRT) can reduce social avoidance for people who experience psychosis, led by Oxford University. The project includes a year-long randomised controlled trial of the gameChange intervention across five NHS trusts. Of 432 participants recruited, half will receive their usual treatment and half will receive six sessions of VRT through a headset guided by a virtual coach. The project also includes a focus on the implementation and adoption of the VRT within the NHS, with involvement a key priority. This poster reports on the implementation strand, led by MindTech in partnership with The McPin Foundation, a mental health research charity.

**Method:**

Barriers and facilitators were identified and, along with the expertise of the project’s Lived Experience Advisory Panel, iteratively informed meetings, workshops and visits involving stakeholders (including staff and service users) in all participating trial sites. The condition, technology, organisation and adopters as well as wider system and value proposition were considered so as to facilitate implementation [1].

**Results:**

The research and design of the VRT was shaped through experiential and professional expertise of the condition and the organisation; through geographical and organisational knowledge accounting for appropriate recruitment and site variability. It also enabled reflection of research practice through prioritisation of data collection methods and analysis, strengthening relevance to real-life practice.

**Conclusion:**

The involvement of potential users from early in development can support not just the intervention’s design but also its delivery and implementation. This enables even new and untried digital health interventions to be designed, developed and delivered in more contextually relevant ways. Consequently, these DHIs can be adopted more confidently into healthcare services.

Thus, we conclude that relevance in practice can come from involvement in research.

**Trial Registration:** ISRCTN17308399

**Reference**

Greenhalgh T, Wherton J, Papoutsi C, Lynch J, Hughes G, Hinder S, Fahy N, Procter R, Shaw S. Beyond adoption: a new framework for theorizing and evaluating nonadoption, abandonment, and challenges to the scale-up, spread, and sustainability of health and care technologies. Journal of medical Internet research. 2017;19(11):e367.

### P15. Factors influencing implementation of two psychoeducational programmes for severe hypoglycaemia in type 1 diabetes: analysis of the qualitative arm of the eeffectiveness-implementation hybrid type 2 trial

#### Tayana Soukup^1^, Louise Hull^1^, Ioannis Bakolis^1^, Andy Healey^1^, Dulmini Kariyawasam^2^, Augustin Brooks^3^, Simon Heller^4^, Stephanie Amiel^5^, Nick Sevdalis^1^, People with Diabetes Group^1^

##### ^1^Centre for Implementation Science, Health Service and Population Research Department, King’s College London, United Kingdom; ^2^Diabetes Department, Guy’s and St Thomas’ NHS Foundation Trust, London, United Kingdom^3^ Diabetes Department, Royal Bournemouth and Christchurch Hospitals NHS Foundation Trust, Dorset, United Kingdom; ^4^Diabetes Department, Sheffield Teaching Hospitals NHS Foundation Trust, Sheffield, United Kingdom; ^5^Diabetes Department, King’s College Hospital NHS Foundation Trust, London, United Kingdom

###### **Correspondence:** Tayana Soukup (Tayana.soukup@kcl.ac.uk)

**Background:**

To facilitate scale-up of two psychoeducational programmes for people with type 1 diabetes and problematic hypoglycaemia, we have set out to build our understanding of facilitators and barriers to their implementation post-trial.

**Method:**

This was an effectiveness-implementation hybrid type 2 trial (NCT02940873) taking place between 2016 and 2021 across five hospitals in the UK and USA. It tested two psychoeducational programmes for managing hypoglycaemia in diabetes.

Qualitative interviews were conducted with 50% of the programme participants (N=41), all healthcare professionals involved in intervention delivery (N=28), and people who declined to take part in the programmes (N=4).

NVivo 12 was used to analyse the interviews and interpret the responses inductively and deductively using a thematic approach. Ethical approval was received and all research participants provided written consent.

**Results:**

Four themes were identified from the interviews as important to consider for scale-up of the two psychoeducational programmes:

1. **Stakeholder buy-in**, incl. both, healthcare professionals and patients, to ensure that sufficient number of patients who would benefit the most from the programmes are identified effectively, given that this is a niche patient group (10% of population with type 1 diabetes),
2. **Adequate funding** to ensure that hospitals in the UK are able to deliver the programmes, and adequate insurance cover is available for the patients in the USA to receive the programme,
3. **Fidelity and quality assurance** to ensure that the programmes are delivered as originally intended providing most benefit to patients;
4. **Adaptations** necessary to increase reach so that more patients have access to the programmes, including flexible mode of delivery, location of the courses, timing of the sessions, and the intensity of the content.

**Conclusion:**

Tension was identified between (1) needing to ensure the fidelity and quality assurance of the programmes post-trial, and (2) adaptations needed to increase reach.

### O16. The role of stakeholder-engaged effectiveness-implementation hybrid designs in health care research: methodological challenges and opportunities through the lens of a case hybrid study

#### Tayana Soukup^1^, Louise Hull^1^, Ioannis Bakolis^1^, Andy Healey^1^, Dulmini Kariyawasam^2^, Augustin Brooks^3^, Simon Heller^4^, Stephanie Amiel^5^, Nick Sevdalis^1^, People with Diabetes Group^1^

##### ^1^Centre for Implementation Science, Health Service and Population Research Department, King’s College London, United Kingdom; ^2^Diabetes Department, Guy’s and St Thomas’ NHS Foundation Trust, London, United Kingdom; ^3^Diabetes Department, Royal Bournemouth and Christchurch Hospitals NHS Foundation Trust, Dorset, United Kingdom; ^4^Diabetes Department, Sheffield Teaching Hospitals NHS Foundation Trust, Sheffield, United Kingdom; ^5^Diabetes Department, King’s College Hospital NHS Foundation Trust, London, United Kingdom

###### **Correspondence:** Tayana Soukup (Tayana.soukup@kcl.ac.uk)

**Background:**

Through the lens of an effectiveness-implementation hybrid type 2 study (NCT02940873) we describe how such multidimensional methodology can help form stakeholder-centred interventions, care and practice. We also reflect on methodological challenges and opportunities with stakeholder-engaged hybrids in the effort to help advance the emerging field of these designs in health care research.

**Method:**

The case study tested two psychoeducational programmes in diabetes. It consisted of effectiveness testing accompanied by the implementation assessments while shaped by the stakeholder inputs - these have been parallel yet mutually interacting formative processes.

The key intervention stakeholders for the hybrid were identified through the stakeholder snowballing technique. The engagement with the identified stakeholders was structured based on the principles reported in the literature, i.e., clear goals for engagement and regular communication channels for continuous partnership building. In total 17 study meetings were conducted with overall 28 intervention stakeholders, including, individuals with lived experience (n=6), and healthcare professionals (n=22).

**Results:**

The stakeholder input has enabled relevant, feasible, and appropriate implementation outcomes, validated surveys, interview questions, participant groups, and measurement time-points to be identified. We also identified key challenges and opportunities of working within such complex research landscape, thus contributing to the scientific understanding of stakeholder-engaged hybrid methods for evaluating and implementing complex interventions within health care. These were as follows: (1) data richness, (2) wide range of participant groups, and (3) pre- and post-intervention assessments.

**Conclusion:**

Stakeholder-led methodology and engagement is critical to ensuring relevance and feasibility of the study design across different hospital settings and countries helping overcome challenges. Such design involves systematic study planning and organisation based on the principles for stakeholder engagement reported in the literature, and a thoughtful assessment of outcomes utilising mixed methods across multi-participant groups and sources.

### P17. Using ‘Theory-of-Change’ methodology to design a scale-up strategy: Lessons from the national implementation of a pragmatic Quality Improvement skills curriculum for urologists

#### Zuhur Balayah^1^, Zarnie Khadjesari^2^, Aoife Keohane^1^, Wilson To^3^, James SA Green^3^, Nick Sevdalis^1^

##### ^1^Center for Implementation Science, Health Service & Population Research Department, Institute of Psychiatry, Psychology & Neuroscience (IoPPN), King’s College London, London, UK; ^2^School of Health Sciences, University of East Anglia, Norwich, UK; ^3^Department of Urology, Bart’s NHS Trust, Whipps Cross Hospital, London, UK

###### **Correspondence:** Zuhur Balayah (zuhur.balayah@kcl.ac.uk)

**Background:**

Engaging stakeholders in some form of ‘co-producing’ a scale-up strategy is often included as a requirement of scale-up efforts. We report an application of stakeholder-driven Theory-of-Change (ToC) methodology to design UK-wide scale-up strategy of an evidenced quality improvement skills training programme for urologists. We narrate the ToC development process; and map the ToC elements onto the Consolidated Framework for Implementation Research (CFIR).

**Method:**

ToC methodology included: (i) multidisciplinary stakeholder input (i.e. urologist consultants/trainees, patients, nurses, managers, medical education/improvement science experts) and (ii) iterative-cycles of defining a consensual scale-up plan. *Phase 1*: national needs assessment and reviews of QI education in surgery (2016-2018) informed draft ToC; *Phase 2*: draft ToC was reviewed in a multidisciplinary workshop (N=10); *Phase 3*: revised ToC was refined in semi-structured stakeholder interviews (N=6) and in a final workshop (N=10). *Phase 4*: refined ToC received a final round of stakeholder interviews (N=4). Data was analysed using the Framework Method.

**Results:**

The ToC (**Figure 1**) specifies assumptions, inputs and activities needed to achieve scale-up, and their enablers. ToC components mapped well onto the CFIR domains: i) identification of underlying assumptions elucidate *intervention characteristics*; ii) implementation strategies listed under activities and associated outcomes link with *characteristics of individuals and process* domains; iii) consensually generated enablers (facilitators/barriers) indicate key features of *inner and outer settings*.

**Conclusion:**

ToC methodology facilitated effective engagement of stakeholders and identification of a nationally implementable scale-up strategy; and allowed mapping of CFIR elements, for subsequent scale-up. We conclude these are complementary approaches for scale-up design.

**Consent to publish**

Not applicable

**Fig. 1 (abstract P17).**
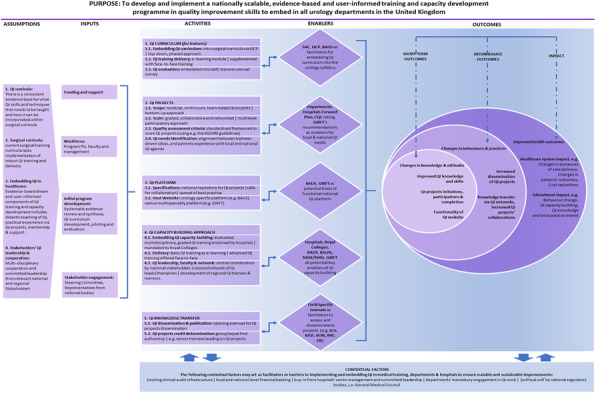
Consolidated Logic Model of implementation plan for education in Quality Improvement (QI) programme for urology residents in the UK, informed by evidence review and key stakeholders’ input (including 3^rd^ sector national representatives from the British Association of Urological Surgeons, BAUS; the British Association of Urological Nurses, BAUN; and The Urology Foundation). Concepts and strategies identified through the application of the Theory-of-Change (ToC) were arranged in a Logic Model. The ToC can be used as an ‘implementation blueprint’ to activate engagement from national bodies to support implementation activities. *Abbreviations:* BJUI = British Journal of Urology International; CQC = Care Quality Commission; GIRFT = Getting It Right First Time; IJUN = International Journal Urological Nursing; ISCP = Intercollegiate Surgical Curriculum Programme; JCU = Journal of Clinical Urology; JME = Journal of Medical Education; JSE = Journal of Surgical Education; NHSE = National Service Health England; NHSI = National Health Service Improvement; SAC = Specialist Advisory Committee

### O18. Combining implementation research with implementation practice to translate evidence-based interventions into routine care within complex adaptive systems: The Systems-informed Participatory Action Implementation Research (SPAIR) approach

#### Hossai Gul^1^, Dr Janet Long^1^, Dr Stephani Best^1,2^, Prof. Frances Rapport^1^, Prof. Jeffrey Braithwaite^1^

##### ^1^Australian Institute of Health Innovation, Macquarie University, Sydney, NSW, Australia; ^2^Murdoch Children’s Research Institute, Melbourne, VIC, Australia

###### **Correspondence:** Hossai Gul (hossai.gul@mq.edu.au )

**Background:**

There is a clinical implementation gap in patient care between what is applied and what we know works best. Changing this within a complex system is challenging. Studying change from outside the system is even harder. This study aimed to develop an anchoring framework seeking to bring implementation research and implementation practice together for the benefit of translating new science into quality care at the coalface of change.

**Method:**

**Phase 1:** Examination of existing literature to understand how researchers outside healthcare approached wicked problems such as poverty, domestic violence, AIDS prevention. Methodological and epistemological aspects of approaches were noted and compared to implementation science models, frameworks, and theories to assess the methodological and epistemological grounding.

**Phase 2:** Development of a new framework for undertaking implementation research in parallel with implementation practice to understand changes in complex adaptive systems.

**Phase 3:** Deployed the newly developed framework across 3 projects focused on the design and implementation of genomic service delivery models.

**Results:**

The new framework - Systems-informed Participatory Action Implementation Research (SPAIR) - combines Systems science, Participatory Action Research (PAR), and Implementation science models, frameworks, and theories (**Figure 1**). Preliminary results using the SPAIR approach: 1) flexible to deploy using implementation frameworks, 2) direct, real-time positive impact on implementation efforts, 3) builds implementation science skills and capacity within organisations, and 4) directly promotes implementation science and practice.

**Conclusion:**

SPAIR can be deployed as the underlying template for leading implementation research and practice within complex adaptive systems.

**Fig. 1 (abstract O18).**
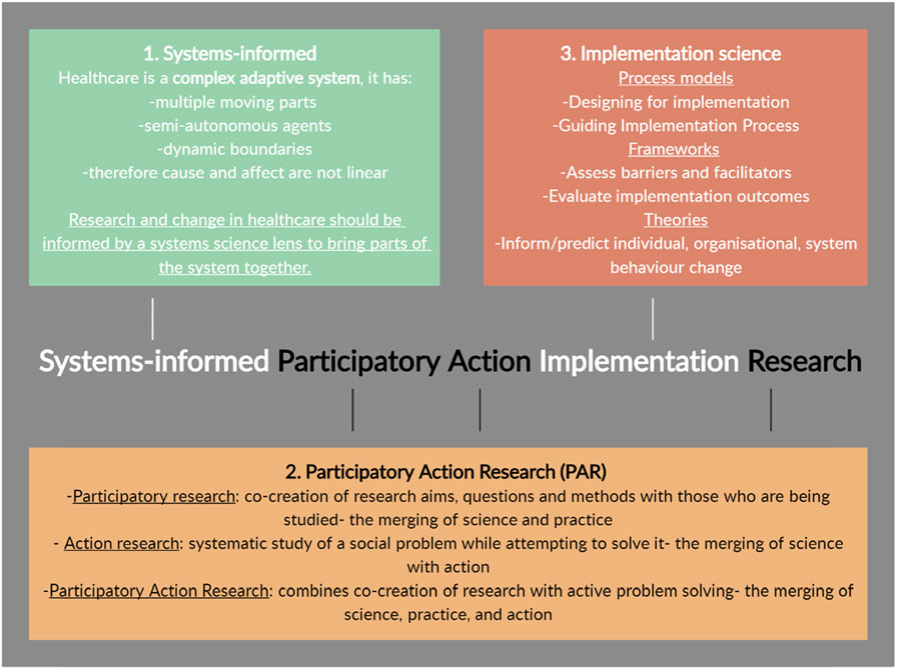
SPAIR framework

### P19. Understanding the intervention co-design process for perioperative antibiotic use at tertiary care hospital in Southern part of India: a two phased qualitative study

#### Shalini Ahuja^1¥^, Gregory Godwin^2¥^, Gabriel Birgand^3^, Andrew Leather^2^, Sanjeev Singh^4^, Pranav V^4^, Nathan Peiffer-Smadja^3^, Esmita Charani^3^, Alison Holmes^3^, Nick Sevdalis^1^, on behalf of co-investigators of ASPIRES

##### ^1^Centre for Implementation Science, Health Services and Population Research Department, King’s College London, UK; ^2^ Faculty of Life Sciences and Medicine, King’s College London, UK; ^3^Faculty of Medicine, Department of Infectious Disease, Imperial College London, UK; ^4^AMRITA Hospital, Kerala, India

###### **Correspondence:** Shalini Ahuja (shalini.ahuja@kcl.ac.uk)

^**¥**^ Joint first authors

**Background:**

Increased antibiotic consumption, linked to antimicrobial resistance and health care associated infections, is a major health issue in low- and middle income countries [1]. Antimicrobial stewardship is a crucial intervention to improve antibiotic usage throughout the surgical pathway and decrease surgical site infection. The aim of this study is to understand the co-design process of selecting interventions and implementation strategies, and to identify barriers and facilitators to the delivery of interventions targeting infection prevention and control (IPC) and antibiotic use perioperatively.

**Method:**

A two-phased qualitative study was undertaken. Phase 1: in depth interviews (n=10) were conducted to understand the context and to identify potential interventions and strategies. Phase 2: theory of change consultative workshops (n=2) explored barriers and facilitators in the implementation of the interventions [2]. Data were analysed using framework thematic analysis and thematic synthesis principles.

**Results:**

Overburdened health workforce along with cultural and professional hierarchies were amongst the various factors identified, exacerbated by organisational factors including lack of resources and ineffective information relay systems. In comparison, existing antimicrobial stewardship (AMS) programme and department specific IPC protocols within the hospital were critical facilitators. Potential implementation strategies were selected: cascade feedback to health workers on infection rates; emphasise AMS and IPC protocols through additional on the job trainings; ensure communication consistency amongst IPC and AMS teams.

**Conclusion:**

Context specific barriers and facilitators can inform implementation practice to reduce inappropriate antibiotic use. Future intervention design studies can consider three policy implications strategies which emerged from our analysis and experience: enhancing consultations during the intervention design, better consideration of implementation challenges during design, and better recognition of co-ordinating mechanisms between different departments.

**References**

1. Charani, E., de Barra, E., Rawson, T. M., Gill, D., Gilchrist, M., Naylor, N. R., & Holmes, A. H. (2019). Antibiotic prescribing in general medical and surgical specialties: a prospective cohort study. Antimicrob Resist Infect Control, 8, 151.

2. Breuer E, Lee L, De Silva M, Lund C. Using theory of change to design and evaluate public health interventions: a systematic review. Implement Sci. 2016;11:63.

### O20. Use of feedback data to reduce surgical site infections and optimise antibiotic use in surgery: a systematic scoping review

#### Shalini Ahuja^1^, Nathan Peiffer-Smadja^3^, Kimberly Peven^2^, Michelle White^2^, Sanjeev Singh^4^, Marc Mendelson^5^, Alison Holmes^3^, Andrew Leather^2^, Gabriel Birgand^3^, and Nick Sevdalis^1^, ASPIRES study coinvestigators

##### ^1^Centre for Implementation Science, Health Services and Population Research Department, King’s College London, UK; ^2^Faculty of Life Sciences and Medicine, King’s College London, UK; ^3^Faculty of Medicine, Department of Infectious Disease, Imperial College London, UK; ^4^AMRITA Hospital, Kerala, India; ^5^Division of Infectious Diseases & HIV Medicine at Groote Schuur Hospital, University of Cape Town (UCT), South Africa

###### **Correspondence:** Shalini Ahuja (shalini.ahuja@kcl.ac.uk)

**Background:**

Surgical site infection (SSI) prevention is a major issue, particularly in the era of antimicrobial resistance. Reducing SSI rates will require, among other priorities, optimisation of antibiotic usage which may be enhanced by feedback [1]. Within the area of surgery, it remains unclear how feedback can best be used to reduce SSIs and improve antibiotic usage. Therefore, this study aims to understand how data from surveillance and audit are utilised in routine surgical practice.

**Method:**

A systematic scoping review was conducted. Two electronic health-oriented databases and the bibliographies of relevant articles were searched. We included studies that assessed the use of feedback as a strategy either in the prevention and management of SSI and/or in the use of antibiotics perioperatively. The results of included studies were synthesised using a narrative synthesis approach underpinning thematic analysis principles. Implementation strategies were grouped into 73 discrete strategies as suggested by the ERIC implementation science research group [2]. The quality of the individual studies was assessed using Integrated Quality Criteria for Systematic Review of Multiple Study Designs.

**Results:**

We identified 21 studies: 17 focused on SSI outcomes and 8 described antibiotic usage in surgery in relation to SSI. These 21 studies described several interventions, mostly multimodal with feedback as a component. Among studies reporting antibiotic usage in surgery most (71%,) discussed compliance with surgical antibiotic prophylaxis. Fifty-five percent of the studies on SSI outcomes reported significant reduction in infection rates. Feedback was often provided in written format (62%), either individualised (38%) or in group (48%). In 65% of the studies, between one and five of 73 ERIC implementation strategies were used while only one study reported using more than 15 implementation strategies.

**Conclusion:**

Our study summarises the efficacy of auditing and surveillance outputs by analysing implementation strategies and highlights the need for feedback to all levels of health care professionals involved in perioperative care of surgical patients.

**References**

1. Biccard, B. M., Madiba, T. E., Kluyts, H.-L., Munlemvo, D. M., Madzimbamuto, F. D., Basenero, A., . . . Zubia, N. Z. (2018). Perioperative patient outcomes in the African Surgical Outcomes Study: a 7-day prospective observational cohort study. *The Lancet, 391*(10130), 1589-1598. doi:10.1016/s0140-6736(18)30001-1

2. Powell, B. J., Waltz, T. J., Chinman, M. J., Damschroder, L. J., Smith, J. L., Matthieu, M. M., . . . Kirchner, J. E. (2015). A refined compilation of implementation strategies: results from the Expert Recommendations for Implementing Change (ERIC) project. *Implement Sci, 10*, 21. doi:10.1186/s13012-015-0209-1

### O21. Deliberation before implementation: Co-designing and co-producing effective relationships in youth justice settings

#### Jackie Dwane, Dr. Sean Redmond, Eoin O’Meara Daly, Caitlin Lewis

##### Research Evidence into Policy, Programme and Practice (REPPP), School of Law, University of Limerick, Limerick, Ireland

###### **Correspondence:** Jackie Dwane (Jacqueline.dwane@ul.ie)

**Background:**

There are 105 youth diversion projects across Ireland targeting young people in trouble with the law. It is estimated that 60 percent of professionals’ time spent in these projects relates to building professional relationships with young people. This relationship effort accounts for approximately €8 million taxpayer’s investment each year. The objective of the relationship is to motivate young people towards pro-social trajectories. However, the practice is largely uncodified or sufficiently described in terms of highlighting and incentivising approaches which are informed by the available evidence.

**Method:**

An Action Research Project (ARP) on behalf of the Department of Justice and Equality is underway to identify the most potent mechanisms within the best relationships. The study involves 16 projects. Initially a Systematic Evidence Review of high quality youth programmes examined underlying relationship ‘mechanisms’. The project then involved academics and practitioners co-designing new evidence informed guidance on relationship-building to improve the effectiveness of everyday practice. An implementation study will complement a realist evaluation of the ARP. The researchers are routinely collecting data through a series of reflective conversations with practitioners over several months to track the experiences of each project implementing the new guidance, time stamped to document key internal and external events. Focused workshops with the wider teams will further interrogate this experience. The researchers are using the Proctor implementation outcomes framework [1] to shape their analysis of the data collected from across the 16 projects.

**Results:**

The implementation study charts the projects’ experience of co-design and transforming guidance into practice. Projects have responded to phase one of the co-design process with enthusiasm and we can report initial ‘buy-in’ and motivation is high.

**Conclusion:**

This presentation will outline the implementation study so far in terms of the methodological design, interim implementation findings, next steps and our reflections on a complex co-design process.

**Acknowledgements**

This study is presented on behalf of Research Evidence into Policy, Programme and Practice (REPPP). The Action Research Project is funded and supported by the Department of Justice and Equality. Special thank you goes to the participating Garda Youth Diversion Projects and the Department of Justice and Equality.

**Reference**

1. Proctor E, Silmere H, Raghavan R, Hovmand P, Aarons G, Bunger A, et al. Outcomes for implementation research: conceptual distinctions, measurement challenges, and research agenda. Adm Policy Ment Health. 2011 Mar;38(2):65–76.

### O22. Accelerating the implementation of evidence: Core competencies for implementation practice

#### Julia E. Moore and Sobia Khan^*^

##### The Center for Implementation, Toronto, Ontario, Canada

###### **Correspondence:** Sobia Khan (Sobia.Khan@thecenterforimplementation.com)

**Background:**

While the field of implementation science has advanced in recent years, this has coincided with a growing divide between the science and practice of implementation. One strategy to bridge this gap is training implementation practitioners to **apply implementation science** to their initiatives in a thoughtful and proactive way. Effective implementation capacity building should be based on core competencies - the knowledge, skills, attitudes, and behaviours needed to apply implementation science. There is a growing body of literature on core competencies for implementation scientists, but the same progress has not been made for core competencies for implementation practitioners. Building applied implementation science capacity at the practitioner level can foster better implementation and overall improved population-level impacts; therefore, understanding the core competencies for applying implementation science at the front line is paramount. The goal of this project was to extrapolate and synthesize core competencies for **implementation practitioners.**

**Method:**

We scanned the published and grey literature to identify core competencies for implementation practice. Six documents outlining (or including components of) core competencies for implementation practice were retrieved. Two analysts reviewed each document using a content analysis approach. Competencies relevant to implementation practice were extracted into an abstraction form and consolidated into a list of common competencies. The refined list of competencies was then grouped thematically into overarching implementation “activities” (e.g., understanding the problem, facilitating implementation).

**Results:**

We identified 37 core competencies which we categorized into 10 implementation activities: Inspiring Stakeholders and Developing Relationships; Building Implementation Teams; Understanding the Problem; Using Evidence to Inform all Aspects of KT; Assessing the Context; Facilitating Implementation; Evaluation; Planning for Sustainability; Brokering Knowledge; and Disseminating Evidence. Additionally, we identified 5 values or guiding principles for implementation practice, which emerged from the document review.

**Conclusion:**

The competencies can be used as a guide to prioritize capacity building efforts.

**Acknowledgements**

Support and funding from Health Canada for this project.

### P23. Pathways to practice change: How to use implementation science theories, models, and frameworks

#### Julia E. Moore and Sobia Khan

##### The Center for Implementation, Toronto, Ontario, Canada

###### **Correspondence:** Julia E. Moore (Julia.moore@thecenterforimplementation.com)

**Background:**

There is recent acknowledgement of a growing divide between implementation science and practice. Unfortunately, applying implementation science in practice can be challenging because implementation scientists have not emphasized how models, theories, and frameworks can be applied. Given that implementation science is an applied science, describing and understanding its real-world applications is critical in order to implement evidence-based interventions and achieve outcomes.

**Method:**

Based on a synthesis of the literature, we developed and administered an approach to train practitioners to apply implementation science to practice.

**Results:**

***Step 1****. Select a process model.* Implementation efforts should first be guided by a process model that describes the actionable steps required to close the evidence-to-practice gap.

***Step 2****. Select a theory of change.* At its core, implementation science is about creating individual, organizational, and/or systems change. Therefore, implementation efforts require a theory of change of each level of change (individual, team, organization, community, system). Theories are typically applied during program development stages of implementation process models, where barriers and facilitators to change are assessed and behavior change strategies are selected that are linked to specific change theories.

***Step 3****. Select frameworks that align with the objectives of each process model stage.* There are over 150 frameworks used in implementation science; therefore, it can be challenging to select an appropriate framework. Most implementation efforts require the use of multiple frameworks, for example a framework to consider individual barriers and facilitators to change, contextual factors, roles in implementation, and implementation outcomes.

**Conclusion:**

This approach to practice implementation provides a roadmap for how to understand and organize the implementation science MTFs in a practical and applied manner. What makes this approach unique is the way that these distinct elements from implementation science, which are inherently interconnected, are linked and woven together to build a practical bridge from research to practice.

### **P24.** From Research to Routine Practice: National Scale Up of a vital signs triage device into routine maternity care in Sierra Leone

#### Alexandra Ridout^1^, Venetia Goodhart^2^, Sophie Bright^2^, Sattu Issa^3^, Betty Sam^2^, Jane Sandall^1^, Andrew Shennan^1^

##### ^1^King’s College London, SE1 7EH, United Kingdom; ^2^Welbodi Partnership, Freetown, Sierra Leone; ^3^Ministry of Health and Sanitation, Freetown, Sierra Leone

###### **Correspondence:** Alexandra Ridout (Alexandra.ridout@kcl.ac.uk)

**Background:**

In Sierra Leone (SL) 1 in 17 women die during pregnancy. The majority of deaths are preventable, detectable by abnormalities in blood pressure and heart rate (vital signs). The CRADLE vital signs monitor is accurate and affordable, incorporating a traffic-light early warning system and shock index calculator.

A hybrid effectiveness-implementation RCT demonstrated that the CRADLE device and training significantly reduced maternal death and eclampsia in SL. Working with the Ministry of Health and Sanitation (MOHS), funded by DfID, we are implementing a national scale-up built on locally piloted strategies.

**Methods:**

The “Theory of Readiness for Change” and “IHI Framework for Going to Full Scale” guided scale-up of this complex intervention. Support systems and adoption mechanisms were continuously iterated. We recorded acceptability, fidelity, adoption and reach alongside policy and practice implications.

**Results:**

MoHS, WHO and UNICEF provided political and organisational leadership alongside key stakeholders at the national launch in January2020. To date 402 MoHS and 23 NGO staff from two districts have been trained. This *Test of Scale* has refined the implementation package across a range of healthcare settings. MoHS have built ownership and sustainability by integrating the programme into SL’s EmONC and Midwifery Schools’ Curricula. The project has benefitted from local redesign, guided by a national working group. The training schedule and device distribution plan have been adapted to align with district health meetings. Whatsapp groups have improved timely data collection and beneficiary feedback.

**Conclusion:**

During this *Test of Scale* we have built a learning system, rolling out 280 CRADLE devices across 139 healthcare facilities and training 402 MoHS healthcare providers. The refined *full scale* is expected to reach 2500 healthcare providers and >750,000 pregnant women in the first year, strengthening the health system capacity, reducing maternal mortality and promoting rollout of the CRADLE package in other countries.

**Trial Registration:** The original CRADLE 3 trial is registered with the ISRCTN registry, number ISRCTN41244132.

### O25. Determinants of keeping the link with Primary Care in a medium-sized city in Brazil

#### Carlos Alberto dos Santos Treichel^1,2^, Ioannis Bakolis^2^, Rosana Teresa Onocko Campos^1^

##### ^1^Department of Collective Health, University of Campinas, Campinas-SP, BR; ^2^Department of Biostatistics and Health Informatics & Health Services and Population Research Department, Centre for Implementation Science, Institute of Psychiatry, Psychology and Neuroscience, Kings College London, London, UK

###### **Correspondence:** Carlos Alberto dos Santos Treichel (treichelcarlos@gmail.com )

**Background**

This study is part of a research that aims to implement devices to integrate the Mental Health Care Network (MHCN) in medium-sized city in Brazil. In order to better inform the implementation process, this study sought to make an evaluative diagnosis about the functioning of the MHCN based on the potential factors of keeping the link with Primary Care (PC) after being on a Specialized Mental Health Care Service (SMHCS).

**Method**

This was a cross-sectional study, conducted with 341 users from SMHCS, between August and November 2019. Crude and adjusted associations between keeping the link with PC after being refereed on a SMHCS and a number of socio-demographic and health care service indicators were explored with the use of Poisson regression models with robust variance estimators.

**Results**

There were strong evidence of a positive association between keeping a link with primary care and have been referred to the current service by the PC (RR: 1.38; 95% CI: 1.06, 1.79); private sector services (RR: 1.66; 95% CI: 1.19, 2.32), being refereed from Community Health Agents (RR: 1.26; 95% CI: 1.04, 1.53). It was less likely to keep the link if you were male (RR: 0.82; 95% CI: 0.67, 0.99), employed (RR: 0.78; 95% CI: 0.60, 1.00), had a diagnosis of psychosis (RR: 0.69; 95% CI: 0.52, 0.91) or substance abuse (RR: 0.57; 95% CI: 0.41, 0.80) and better social support (RR: 0.73; 95% CI: 0.58, 0.91).

**Conclusion**

While there is a gap regarding keeping the link with PC among users diagnosed with psychosis and substance abuse, the action of the PC in the referral of cases and follow-up through the visit of Community Health Agents proved to be a strategy that should be encouraged in order to promote the keeping of the link with PC among users of the MHCN.

**Acknowledgements**

This study was financed in part by the Coordenação de Aperfeiçoamento de Pessoal de Nível Superior - Brasil (CAPES) - Finance Code 001. The research that originated this study was funded by the Fundação de Amparo à Pesquisa do Estado de São Paulo (FAPESP), process nº 2018/10366-6.

### P26. Evaluation of Project Echo: Palliative care education for nurses in long term care facilities

#### Alice Coffey^1^, Helen Flanagan^2^, Martina O’Reilly^2^, Valerie O’Reilly^2^, Pauline Meskell^1^, Maria Bailey^1^, Eileen Carey^1^, Jane O’Doherty^1^, Cathy Payne^3^, Karen Charnley^3^

##### ^1^Department of Nursing and Midwifery, University of Limerick, Limerick, Ireland; ^2^Milford Care Centre, Limerick, Ireland; ^3^All-Ireland Institute of Hospice and Palliative Care, Dublin, Ireland

###### **Correspondence:** Jane O’Doherty (Jane.ODoherty@ul.ie)

**Background:**

Project ECHO is a pioneering tele-mentoring programme, which was developed in the School of Medicine at the University of New Mexico. It uses technology and existing resources to magnify the capacities of the health care workforce, building a bridge across healthcare settings, and providing healthcare without barriers. The model was shown to be an effective way of addressing the knowledge gap among healthcare professionals. The aim of this study was to evaluate the impact of Phase 3 of Project ECHO.

**Methods:**

A mixed method approach underpinned by the RE-AIM framework (Reach, Effectiveness, Adoption, Implementation and Maintenance) [1]. Data were collected from participating sites on resident referral, evaluation of educational sessions and focus groups with ECHO participants.

**Results:**

Reach:15 nursing homes were invited to take part and 12 participated. Efficacy: Data from the ECHO session evaluations and focus groups showed a positive impact on participants, particularly from the shared learning experiences but areas for future learning were identified. Adoption: Staff reported knowledge improvement and the translation into changes in practice. Implementation: Data on resident referral to acute care during the ECHO project shows that 50% were referred by nursing home staff.

**Conclusions:**

Recommendations for maintenance of positive outcomes include strategies to improve staff participation and encourage General Practitioner involvement in the programme.

**Acknowledgements**

The authors wish to thank the Health Service Executive (HSE) who commissioned this work for use by All-Ireland Institute of Hospice and Palliative Care (AIIHPC). Thanks to all nursing home staff, nursing home managers, education providers and specialist palliative care team members who have taken part in this research.

**Reference**

Green LW, Glasgow R. Evaluating the Relevance, Generalization, and Applicability Of Research. Eval Health Prof. 2006; 29:(1), 126-153.

### O27. Furthering implementation science in HIV research through coordination of, consultation with, and collaboration among 65 projects to end the HIV epidemic in the US

#### Dennis H Li^1,2,3,4^, Nanette Benbow^1,2,3^, JD Smith^1,2,3,5,6^, Juan Villamar^1,2^, Brennan Keiser^4^, Melissa Mongrella^4^, Thomas Remble^2,4,5^, Brian Mustanski^1,2,3,4,5^

##### ^1^Department of Psychiatry and Behavioral Sciences, Northwestern University, Chicago, IL, USA; ^2^Center for Prevention Implementation Methodology, Northwestern University, Chicago, IL, USA; ^3^Third Coast Center for AIDS Research, Chicago, IL, USA; ^4^Institute for Sexual and Gender Minority Health and Wellbeing, Northwestern University, Chicago, IL, USA; ^5^Department of Medical Social Sciences, Northwestern University, Chicago, IL, USA; ^6^Department of Preventive Medicine, Northwestern University, Chicago, IL, USA

###### **Correspondence:** Dennis H Li (dennis@northwestern.edu )

**Background:**

The first year of the USA’s *Ending the HIV Epidemic* (EHE) strategy funded 65 planning projects in 46 high-priority jurisdictions to begin studying implementation of evidence-based HIV interventions in local healthcare and public health systems. To maximize the value of implementation science (IS) in these projects, we established the Implementation Science Coordination, Consultation, and Collaboration Initiative (ISC^3^I) with two goals: (1) support high-quality IS through expert technical assistance and (2) create opportunities to develop generalizable knowledge from local knowledge through cross-project information sharing, measure harmonization, and data synthesis. This presentation describes the first year of this innovative approach to coordinating HIV implementation research nationally.

**Methods:**

To launch ISC^3^I, we invited project leads, their primary implementation partners, and federal health agencies to a two-day summit that focused on applying IS concepts to HIV contexts, facilitated researchers and partners’ co-development of an implementation research logic model, and fostered cross-project dialogue. We created an online community of practice (COP) as a clearinghouse for IS resources and ISC^3^I training and collaboration activities (e.g., webinars, expert coaching, videoconference discussions). We also established infrastructure to collect data from the projects, which we are using to inform coordinated IS measures and constructs to put forth for recommended use across future EHE-related projects.

**Results:**

Because most project leads had limited prior IS training or experience, and most projects are in the formative stage, ongoing coordination challenges include differentiating interventions from implementation strategies and identifying appropriate implementation outcomes. However, many teams have engaged with ISC^3^I activities and reported them to be helpful. Additional lessons learned will be discussed.

**Conclusions:**

ISC^3^I represents an unprecedented opportunity to expand IS capacity and develop generalizable knowledge for HIV prevention and treatment in the US. We aim to further codify our measure harmonization efforts as we move into the next year of EHE.

### P28. Implementer and researcher perspectives on sustaining, spreading and scaling up, quality improvement interventions

#### Celia Laur^1^, Ann Marie Corrado^1^, Jeremy Grimshaw^2^, Noah Ivers^1^

##### ^1^Women’s College Hospital, Toronto, Ontario, M5S 1B2, Canada; ^2^Ottawa Hospital Research Institute, Ottawa, Ontario, K1Y 4E9, Canada

###### **Correspondence:** Celia Laur (Celia.Laur@wchospital.ca)

**Background:**

Quality Improvement (QI) programs rarely consider how their intervention can be sustained long-term. Failing to adequately consider sustainability contributes to research waste and has the potential to make patient outcomes worse, if patients relied upon the QI program to improve quality of care. A survey of authors of randomized trials of diabetes QI interventions included in an ongoing systematic review found that 78% of trials reported improved quality of care, but 40% of these trials were not sustained following study completion. This study further explores why and how the effective interventions were sustained, spread or scaled.

**Method:**

This study features telephone interviews with those who have implemented diabetes QI intervention studies between 2004-2014 included in a systematic review, completed the sustainability survey, and agreed to further contact. Two team members independently used inductive coding to identify key themes, with case examples used to show trajectories across projects and people.

**Results:**

Eleven trial authors (n=9 male; 13 studies) participated. 12/13 studies featured interventions that were deemed “effective” in the survey; 5/13 reported that the intervention was “sustained”. Four interacting themes were identified: understanding the *concepts* of implementation, sustainability, sustainment, spread and scale; knowing the roles of the *people* involved; having the appropriate *competencies*; and that individual and organisational *capacity* is needed. Participant stories highlight the varied *trajectories* of projects and people, such as the participant who led an effective intervention, however left academia in order to implement it at scale.

**Conclusion:**

Researchers need to think beyond effectiveness and consider if an intervention is also feasible and sustainable, with potential for spread or scalability. Lessons learned highlight the potential for collaborating with experts outside of health, such as those with expertise in business and organizational management.

### P29. Application of the Pragmatic-Explanatory Continuum Indicator Summary tool (PRECIS-2) to two delivery strategies of an eHealth HIV prevention intervention in a hybrid comparative implementation trial

#### N. Benbow^1^, K. Macapagal^2^, J. Jones^2^, K. Madkins^2^, J.D. Smith^1^, D. H. Li^1^, B. Mustanski^2^

##### ^1^Northwestern University Feinberg School of Medicine, Department of Psychiatry and Behavioral Sciences, Chicago, IL 60611, United States; ^2^Northwestern University Feinberg School of Medicine, Medical Social Sciences, Chicago, IL 60611, United States

###### **Correspondence:** N. Benbow (Nanette@northwestern.edu)

**Background:**

There is still much to understand about scaling-up eHealth HIV prevention interventions. Implementation research can help bridge the research-to-practice gap, but study conditions must emulate real-world delivery contexts and procedures to maximize knowledge gained. In the context of a randomized comparative implementation trial, we evaluated the pragmatism of two delivery approaches for an evidence-based eHealth HIV prevention intervention for young men who have sex with men.

**Method:**

Keep It Up! is an effectiveness-implementation type III hybrid cluster RCT comparing two strategies designed to resemble real-world implementation: direct-to consumer (DTC) vs. delivery through community-based organizations (CBO). Using the Pragmatic-Explanatory Continuum Indicator Summary tool (PRECIS-2) [1], we compared the strategies on nine domains, each scored on a continuum (1=very explanatory to 5=very pragmatic). Three coders per arm ranked and provided rationale for each domain and discussed differences to arrive at consensus ranking. Results were reviewed with three additional coders to ensure coding standardization across arms.

**Results:**

Both arms ranked as rather or very pragmatic on most domains. They had equal scores on seven of nine domains: eligibility, recruitment, flexibility of intervention in delivery and adherence, follow-up, primary outcome, and primary analysis. CBO delivery scored lower than DTC on the setting domain due to lower-than-realistic funding for CBO implementation. DTC delivery scored lower than CBO on the organization domain based on staff’s expertise and resources delivering DTC that may not be matched under usual conditions. Interpretation of some PRECIS-2 domains varied between arms, where DTC focused on individual users of the intervention and CBO focused on implementers.

**Conclusion:**

Application of the PRECIS-2 helped validate our pragmatic study design and increased our confidence that both arms highly resembled real-world implementation procedures. This is one of the few applications of PRECIS-2 to an implementation trial and highlights the need for minor modifications to the tool for this purpose.

**Reference**

1. Loudon K, Treweek S, Sullivan F, Donnan P, Thorpe K, Zwarenstein M. The PRECIS-2 tool: designing trials that are fit for purpose. BMJ 2015;350:h2147

### O30. Implementation science in marginalised poorly resourced communities: a case study using the Nurture Early for Optimal Nutrition (NEON) intervention to improve infant feeding, care and dental hygiene practices in South Asian infants aged < 2 years in East London

#### Dr Logan Manikam^1,2^, Shereen Allaham^1,2^, Dr Michelle Heys^3^, Dr Clare Llewellyn^4^, Dr Neha Batura^5^, Prof Andrew Hayward^1^, Yasmin Bou Karim^2,3^, Jenny Gilmour^6^, Kelley Webb-Martin^7^, Carol Irish^7^, Chanel Edwards^7^, Prof Monica Lakhanpaul^3,8^

##### ^1^Department of Epidemiology and Public Health, UCL Institute of Epidemiology and Health Care (IEHC), London, UK; ^2^Aceso Global Health Consultants Ltd, London, UK; ^3^ Population Policy and Practice (PPP), UCL Great Ormond Street Institute of Child Health (GOS-ICH), London, UK; ^4^Department of Behavioural Sciences & Health, UCL IEHC, London, UK; ^5^UCL Institute for Global Health, London, UK; ^6^Tower Hamlets GP Care Group, Mile End Hospital, London, UK; ^7^Children’s Health 0-19 Service, Newham Council, London, UK; ^8^ Whittington Health NHS Trust, London, UK

###### **Correspondence:** Logan Manikam (logan.manikam.10@ucl.ac.uk)

**Background:**

The first 1,000 days of a child’s life are an important period for growth and cognitive development. Exposures during pregnancy and infancy may alter lifetime risk of overall development and dental health [1]. The Participatory Learning and Action (PLA) is a low-cost bottom-up approach that mobilises communities to identify, prioritise, implement, and evaluate their needs and solutions through culturally-sensitive group discussions [2]. Recognising PLA has been successful in LMICs and the importance of community engagement, the NEON study aimed at improving infant feeding, care, and dental hygiene practices of South Asians (SA) in two deprived East London boroughs (Tower Hamlets & Newham) by reverse innovating the WHO-recommended PLA approach from LMICs.

**Method:**

Our approach was developed through a series of workshops with community and local stakeholders facilitated by experts in PLA. Adaptation is supported by multilingual community facilitators (CFs) and the local health and social care systems. We are currently co-developing the PLA intervention toolkit consisted of; (i) PLA group facilitator manual, (ii) picture cards, (iii) healthy food recipes & (iv) community asset map by undertaking monthly development meetings with SA CFs (n=10) and refinement workshops with a larger audience of SA residents (n=50). Initially done face-to-face, we are now utilising blended-approach of online meetings due to COVID-19.

**Results:**

The PLA approach was highly acceptable to participants. However, the feasibility of undertaking 12-session PLA cycle was questioned. We have since adapted the model to shorter cycles (7&6 session). Strong community ownership presented with CFs engaged in developing culturally-tailored PLA intervention content including a digitally shareable asset map consisting of local resources and services.

**Conclusion:**

NEON is an exemplar of how to adapt tailored culturally-sensitive community-based intervention from LMICs to urban high-income setting. The PLA is an acceptable and feasible approach to address public health issues in marginalised poorly-resourced and ethnically-diverse community.

**References**

1. Aris IM, Bernard JY, Chen LW, Tint MT, Pang WW, Soh S-E, et al. Modifiable risk factors in the first 1000 days for subsequent risk of childhood overweight in an Asian cohort: significance of parental overweight status. International Journal of Obesity. 2018;42(1):44.

2. Freire P. Education for critical consciousness: Bloomsbury Publishing; 1973.

### O31. Pragmatic parallel mixed methods investigation into implementation of home-based cardiac rehabilitation for heart failure patients: methodological discussion

#### Paulina Daw ^1^, Dr Jet Veldhuijzen van Zanten^1^, Alexander Harrison^2^, Dr Hasnain Dalal ^3,4^, Prof Rod S Taylor ^5,3^, Prof Patrick J Doherty ^2^ , Dr Sinead TJ McDonagh^3^, Prof Colin J Greaves^1^

##### ^1^School of Sport, Exercise & Rehabilitation Sciences, University of Birmingham, UK; ^2^Health Sciences, University of York, UK; ^3^University of Exeter Medical School, Exeter, UK; ^4^Royal Cornwall Hospitals NHS Trust, Cornwall, UK; ^5^MRC/CSO Social and Public Health Sciences Unit & Robertson Centre for Biostatistics, Institute of Health and Well Being, University of Glasgow, Glasgow, UK

###### **Correspondence:** Paulina Daw (pxd891@student.bham.ac.uk)

**Background:**

Low uptake of cardiac rehabilitation (CR) is a global problem, particularly for heart failure patients (HFPs), who are often older and more frail than other cardiac patients. Offering alternative forms of CR (e.g. home-based programmes) might improve uptake. This is especially relevant in the current COVID-19 pandemic where HFPs are asked to self-isolate.

Rehabilitation Enablement in Chronic Heart Failure (REACH-HF) is a home- and evidence-based CR programme for HFPs. The effectiveness of REACH-HF has been established in two clinical trials.[1,2] Subsequently, four NHS Beacon Sites are delivering REACH-HF to 200 patients in England and Northern Ireland.

**Method:**

A multi-method study, conducted in five phases (two completed and three ongoing), to evaluate the implementation of REACH-HF. The Normalisation Process Theory will be used as a framework to inform data collection/analysis.[3]

1. A systematic review identified professional and system-level factors affecting the delivery of CR for HFPs.

2. Qualitative in-depth interviews and focus groups with key healthcare professionals (HCPs). Thematic analysis of qualitative data informed a pragmatic REACH-HF implementation manual for HCPs.

3. Participatory action research: feedback from key stakeholders will lead to refinement of the implementation manual. This will be further piloted in an implementation study in Scotland.

4. Implementation fidelity will be assessed by coding audio recordings of treatments using the existing REACH-HF fidelity checklist. Scores will be compared to fidelity achieved in the clinical trial.

5. Audit: pre-post treatment outcomes will be analysed using data from the National Audit of Cardiac Rehabilitation. Real-world patient outcomes will be compared to outcomes achieved in the clinical trial.

**Results:**

Summary data will be presented from the systematic review and qualitative elements of the study, along with a discussion of the planned synthesis of data from all five phases using meta-ethnography.[4]

**Conclusion:**

When completed, this study will identify ways to improve the CR provision for HFPs.

**Acknowledgements**

REACH-HF Beacon Sites Steering Group

**References**

1. Dalal H., Taylor R., Jolly K., et al. The effects and costs of home-based rehabilitation for heart failure with reduced ejection fraction: The REACH-HF multicentre randomized controlled trial. Eur J Prev Cardiol. 2018;27:3.

2. Lang C., Smith K., Wingham J., et al. A randomised controlled trial of a facilitated home-based rehabilitation intervention in patients with heart failure with preserved ejection fraction and their caregivers: the REACH-HFpEF Pilot Study. BMJ Open. 2018;8:4.

3. May C., Mair F., Finch T., et al. Development of a theory of implementation and integration: Normalization Process Theory. Implement. Sci. 2009;4:29 (2009)

4. Noblit G., Hare R. Meta-ethnography: synthesizing qualitative studies. Volume 11. California: Sage Publications;1988.

### O32. Return on investment analysis of nationwide implementation of the WHO Surgical Safety Checklist in Madagascar, Benin and Cameroon

#### Michelle C White^1,2,3^, Andrew JM Leather^1^, Nick Sevdalis^4^, Andy Healey^4,5^

##### ^1^Centre for Global Health and Health Partnerships, King’s College London, London, SE5 9RJ, United Kingdom; ^2^Great Ormond Street Hospital for Children, London, WC1N 3JH, United Kingdom; ^3^Mercy Ships UK, The Lighthouse, Stevenage, SG1 2EF, United Kingdom; ^4^Centre for Implementation Science, Health Service and Population Research Department, Institute of Psychiatry, Psychology and Neuroscience, King’s College London, London, SE5 8AB, United Kingdom; ^5^King’s Health Economics, Health Service and Population Research Department, Institute of Psychiatry, Psychology and Neuroscience, King’s College London, London, SE5 8AB, United Kingdom

###### **Correspondence:** Michelle C White (doctormcw@gmail.com)

**Background:**

Limited resources in Low and Middle-Income Countries (LMICs) mean health interventions must compete against other projects for political priority. Policy-makers make decisions using subjective and objective criteria [1]. The World Health Organisation (WHO) Surgical Safety Checklist reduces surgical mortality and morbidity [2] but its economic impact is unknown. We undertook a return-on-investment (ROI) analysis of checklist scale-up in Madagascar, Benin and Cameroon.

**Method:**

The ROI analysis used two approaches: cost-effectiveness analysis (CEA) and benefit-cost analysis, (BCA). We estimated the years of life lost (YLL) due to post-operative mortality that would be averted through checklist use, and then using total project costs we estimated incremental cost-effectiveness ratios (ICER) for each country. We estimated benefit-cost ratios (BCR) using the value of a statistical life-year approach. All monetary values are expressed in US$ using World Bank purchasing power parity and discounted at 4%.

**Results:**

The ICERs are $62, $16 and $74 per YLL averted, and the BCRs are 17, 120 and 55 for Madagascar, Benin and Cameroon, respectively. The BCRs mean that for every $1 USD spent, the potential return is $17 – 120. Following WHO criteria [3] checklist scale up is ‘very cost-effective’; using more stringent criteria, it ranks within the top 33 cost-effective interventions in LMICs (see Table 1) [4].

**Conclusion:**

Checklist scale-up is very cost-effective and gives a good ROI. This research offers policy-makers evidence to help make checklist implementation a national priority. Our methodology offers a ‘blueprint’ for including implementation costs in health economic evaluations of other safety and quality improvement interventions.

**Acknowledgements**

The authors wish to thank Mercy Ships for undertaking the WHO checklist implementation and providing a detailed breakdown of implementation costs.

**References**

1. Shiffman J, Smith S Generation of political priority for global health initiatives: a framework and case study of maternal mortality Lancet 2007: 370; 1370-1379

2. Bergs J, Hellings J, Cleemput I*, et al.* Systematic review and meta-analysis of the effect of the World Health Organization surgical safety checklist on postoperative complications Br J Surg 2014: 101; 150-158

3. WHO-CHOICE: CHoosing Interventions that are Cost–Effective, Geneva, World Health Organisation, 1998.

4. Horton S, Gelband H, Jamison D*, et al.* Ranking 93 health interventions for low- and middle-income countries by cost-effectiveness PloS one 2017: 12; e0182951

**Table 1 (abstract O32).** Incremental cost effectiveness ratios (ICER) and benefit-cost ratios (BCR) of health and non-health interventions

**Incremental cost effectiveness ratios of surgical interventions and compared with other public health interventions (cost in USD per DALY or YLL averted)**			
**Surgical Intervention**	**ICER**	**Other health intervention**	**ICER**
Male circumcision	$7- $106	Vitamin A supplementation	$6-$12
***WHO checklist scale-up***	***$16 - $74***	BCG vaccination	$51 - $220
Cleft lip and palate surgery	$15 - $96		
General surgery	$82		
Hydrocephalus surgery	$108		
Ophthalmic Surgery	$136		
Orthopaedic surgery	$381	Anti-retroviral therapy for HIV	$453 - $648
		Medical therapy for ischaemic heart disease	$500 - $706
Caesarean Section	$315	Breast-feeding promotion	$930
		Oral rehydration solution therapy	$1062
**Benefit cost ratios of health and non-health Interventions**			
**Intervention**		**Benefit-cost ratio**	
Caesarean section, globally		4	
Essential surgical procedures, globally		10	
Cleft lip and palate repair, India		14	
Investment to retrofit schools in India to better withstand earthquakes, India		0.04 – 5.6	
***WHO checklist scale-up***		***17-120***	
reducing the prevalence of stunting by a package of interventions targeting malnutrition, India		44 – 138	

### P33. RECODE-DCM Enviro-Scan: Identifying key agents of change for implementation of findings from a James Lind Alliance priority partnership

#### Ben Grodzinski^1^, Harry Bestwick^1^, Faheem Bhatti^1^, Rory Durham^1^, Maaz Khan^1^, Celine Partha-Sarathi^1^, Jye Quan Teh^1^, Oliver Mowforth^2^, Benjamin M. Davies^2*^ (On behalf of AO Spine RECODE-DCM Consortia)

##### ^1^School of Clinical Medicine, University of Cambridge, Cambridge, UK; ^2^Academic Neurosurgery Unit, Department of Clinical Neurosurgery, University of Cambridge, Cambridge, UK

###### **Correspondence:** Ben Grodzinski (bd375@cam.ac.uk)

**Background:**

Degenerative Cervical Myelopathy (DCM) is a progressive disorder of the spinal cord, caused by arthritis. It is common (~2% of adults) and has a significant impact on health-related quality of life. Advances in care are urgently needed.

RECODE-DCM (*Re*search Objectives and *Co*mmon *D*ata *E*lements in DCM) is an international collaboration which aims to improve research efficiency in DCM, and ultimately accelerate advances in care. As a first step, it has recently completed a James Lind Alliance research priority-setting process.

RECODE’s success now depends on the dissemination and uptake of these priorities. However, this is challenging in DCM for a number of reasons, including a lack of recognition, lack of established lobbying or advocate groups, and the numerous different healthcare professionals involved in management pathways.

**Method:**

To facilitate implementation key stakeholders termed ‘Agents of Change’ (AoC) were identified at a multi-disciplinary workshop. A tailored international search strategy was then conducted to identify potential agents, itemising with key meta-data and indexing against relevant research priorities.

**Results:**

Researchers, funders, non-profit and charities and scientific conferences were identified as key AoC. These were identified in three project arms. The research arm created a database of conferences at which to promote the research priorities, a database of researchers already working on the priorities, and a database of journals in which this work is being published. The funding arm created a database of current and potential funders. The organisations arm created a database of charities and non-profit organisations which could help promote the priorities. Project completion date 20th May 2020.

**Conclusion:**

Research on DCM, whilst currently under-developed, is rapidly accelerating. The RECODE-DCM Enviro-Scan has identified and indexed key agents for its implementation.

### O34. A co-designed intervention to enhance the national audit of dementia

#### Michael Sykes^1^, Richard Thomson^1^, Niina Kolehmainen^1^, Louise Allan^2^, Tracy Finch^3^

##### ^**1**^Population Health Sciences Institute, Newcastle University, Newcastle upon Tyne, NE2 4AX, UK; ^2^College of medicine and health, University of Exeter, Exeter, EX1 2LU, UK; ^3^Department of Nursing, Midwifery & Health, Northumbria University, Newcastle upon Tyne, NE7 7XA, UK

###### **Correspondence:** Michael Sykes (michael.sykes@ncl.ac.uk)

**Background:**

Patients with dementia do not always get best care [1]. Hospitals use audit and feedback to improve dementia care. Audit and feedback is variably effective at improving care [2]. There have been calls to test potential enhancements to national audit [3]. Both evidence and theory describe practices that might affect the effectiveness of audit and feedback [2,4]. We aimed to describe the content and delivery of the national audit of dementia, identify potential enhancements and develop a strategy to implement the enhancements.

**Method:**

We purposively sampled six hospitals, semi-structured interview participants (n=32), observations (n=36) and documentary analysis (n=39). We used framework analysis.

Interim analysis was iteratively presented to stakeholders during co-design workshops (n=9; 18 hours) for challenge and to integrate findings, until a stable description was developed. The co-design group specified potential enhancements (3 workshops; 6 hours). Further co-design workshops (n=2; 4 hours) used the normalisation process theory toolkit [5] to identify mechanisms affecting implementation. This analysis informed a specified [6] implementation strategy.

**Results:**

Hospital actions were not informed by a robust analysis of performance, were selected from a narrow range of implementation strategies [7] and were not presented in a way to gain organisational commitment [8]. We co-designed a training intervention to hospital dementia leads and clinical governance leads that aims to improve the development and agreement of hospital-level actions. The intervention trains the leads to present information which supports governance committee sense-making in relation to implementation capability (by targeting low baseline, analysing barriers and linking barriers to actions) and change commitment (by addressing trust and credibility, linking to priorities, presenting comparators and considering existing work) [2,3,7,8].

**Conclusion:**

Training clinical leads to analyse performance, investigate barriers, select strategies and present specific information designed to gain organisational commitment may enhance the effectiveness of the national audit of dementia.

**Acknowledgements**

This work is being presented on behalf of the co-design group members, without whom this work would not have been possible.

**Consent to publish**

All participant gave their consent for material to be used in this publication.

**References**

[1] Royal College of Psychiatrists. National Audit of Dementia care in general hospitals 2018-2019: Round four audit report. London: Royal College of Psychiatrists. 2019.

[2] Ivers N, Jamtvedt G, Flottorp S, Young JM, Odgaard‐Jensen J, French SD, et al. Audit and feedback: effects on professional practice and healthcare outcomes. Cochrane database of systematic reviews. 2012. 6.

[3] Colquhoun HL, Carroll K, Eva KW, Grimshaw JM, Ivers N, Michie S, et al. Advancing the literature on designing audit and feedback interventions: identifying theory-informed hypotheses. Implement Sci. 2017;12(1):117.

[4] Grimshaw JM, Ivers N, Linklater S, Foy R, Francis JJ, Gude WT et al. Reinvigorating stagnant science: implementation laboratories and a meta-laboratory to efficiently advance the science of audit and feedback. BMJ Qual Saf. 2019 May 1;28(5):416-23.

[5] May CR, Finch T, Ballini L, MacFarlane A, Mair F, Murray E, Treweek S, Rapley T. Evaluating complex interventions and health technologies using normalization process theory: development of a simplified approach and web-enabled toolkit. BMC health serv res. 2011;1;11(1):245.

[6] Michie S, Richardson M, Johnston M, Abraham C, Francis J, Hardeman W, Eccles MP, Cane J, Wood CE. The behavior change technique taxonomy (v1) of 93 hierarchically clustered techniques: building an international consensus for the reporting of behavior change interventions. Ann behav med. 2013;1;46(1):81-95.

[7] Powell BJ, Waltz TJ, Chinman MJ, Damschroder LJ, Smith JL, Matthieu MM, Proctor EK, Kirchner JE. A refined compilation of implementation strategies: results from the Expert Recommendations for Implementing Change (ERIC) project. Implement Sci. 2015; 1;10(1):21.

[8] Weiner BJ. A theory of organizational readiness for change. Implement Sci. 2009;1;4(1):67.

### O35. Implementing the HOUVAST 2.0 intervention in the Dutch primary care; lessons learned from a process evaluation

#### S. Hogervorst^1^, M.C. Adriaanse^1^, H.E. Brandt^1^, M. Vervloet, L^3^. van Dijk^3,4^, J.G. Hugtenburg^2^

##### ^1^Department of Health Science and the Amsterdam Public health Institute, Vrije Universiteit, Amsterdam, The Netherlands; ^2^Department of Clinical Pharmacology and Pharmacy, Amsterdam UMC, loc. VUmc, Amsterdam, The Netherlands; ^3^Netherlands institute for health services research (NIVEL), Utrecht, The Netherlands; ^4^Faculty of Science and Engineering, University of Groningen, The Netherlands

###### **Correspondence:** S. Hogervorst (s.hogervorst@vu.nl)

**Background:**

Despite the existence of many effective adherence interventions, they are rarely used in routine care. This gap between research and practice calls for more emphasis on the implementation of adherence interventions. This pilot project aims to implement an existing adherence intervention (HOUVAST 2.0) in the Dutch primary care.

**Method:**

A qualitative process evaluation was conducted as part of a medication adherence pilot project (HOUVAST 2.0). Data were collected through a focus group and four interviews with ten allied GP and pharmacy staff members. Interviews and focus groups were semi-structured using topic lists based on the RE-AIM implementation framework. Interviews were audiotaped and transcribed verbatim. Atlas.ti 8.0 software was used for coding and structuring of themes. A thematic analysis of the data was performed.

**Results:**

Main themes that emerged were ‘Training and preparation’, ‘Appreciation for the intervention’ ‘Technical barriers to implementation’ and ‘social barriers to implementation’.

The intervention HOUVAST 2.0 proved engaging for clinicians that used the intervention, but also proved difficult to implement. The main barriers were a suboptimal selection process based on pharmacy refill data, a difficult target population, nurse practitioners’ difficulties addressing adherence with patients and the project did not align with goals of GPs.

**Conclusion:**

Implementation of the HOUVAST 2.0 intervention in the Dutch primary care proved challenging. A good established collaboration between GPs and pharmacies, better ICT applications for selecting patients and a training more aimed towards practical communication techniques are important improvements needed for further implementation.

**Consent to publish**

Oral informed consent from all patients and healthcare providers to anonymously record and analyse the data was obtained before conducting the interviews/focus groups

### P36. Improving the quality of nursing documentation for surgical patients in a referral hospital in Freetown, Sierra Leone: a study protocol of a multifaceted quality improvement project

#### Nataliya Brima^1^, Nick Sevdalis^2^, TB Kamara^3^, H Wurie^4^, K Daoh^5^, B Deen^5^, Justine Davies^6^^ and Andrew Leather^1^^

##### ^1^King’s Centre for Global Health & Health Partnerships, School of Population Health & Environmental Sciences, Faculty of Life Sciences and Medicine, King’s College London, London, UK; ^2^Centre for Implementation Science, Health Service and Population Research Department, Kings College London, UK; ^3^Department of Surgery, College of Medicine and Allied Health Sciences, University of Sierra Leone, Freetown, Sierra Leone; ^4^Faculty of Nursing, College of Medicine and Allied Health Sciences, University of Sierra Leone, Freetown, Sierra Leone; ^5^Connaught Teaching Hospital Complex, Sierra Leone, Freetown, Sierra Leone; ^6^Institute of Applied Health Research, University of Birmingham, UK

###### **Correspondence:** Nataliya Brima (nataliya.brima@kcl.ac.uk)

^ co-senior authors

**Background:**

The global health community is placing greater emphasis on quality of care, while not neglecting access to care, in order to reduce avoidable mortality and morbidity from surgical diseases in low- and middle-income countries. However, many of these health systems are weak and provide low quality health care. There is a lack of knowledge on how health system strengthening quality improvement interventions can be implemented effectively in these settings. To address this gap, we developed a multifaceted quality improvement project to improve the quality of nursing documentation, through implementation and evaluation of a set of hospital-based activities.

**Methods:**

This multifaced quality improvement, mixed-method, quasi-experimental design interventional study has been co-designed during an intensive formative phase guided by a theory of change process. It will take place within the surgical department of a national referral hospital in Freetown, Sierra Leone.

The study is structured around five distinct phases – pre-implementation, awareness drive, training package, audit and feedback, and evaluation. Plan-Do-Study-Act quality improvement method will be used to provide further evidence to optimise the set of interventions and implementation strategies.

**Results:**

The primary outcome of the study is composite measure of completeness of the Nurses Daily Report form. In addition, several process and implementation outcomes will be evaluated to study effects of interventional components and implementation strategies. Further information on sustainability of nursing documentation quality improvement processes will also be collected.

**Conclusion:**

We seek to test if the quality of nursing documentation can be improved through the introduction of a set of health system strengthening interventions, using implementation and improvement sciences methods.

The results will generate knowledge to inform good nursing documentation practices for surgical patients in Sierra Leone, add to the body of evidence on the development and implementation of effective health system strengthening quality improvement interventions in low resource settings.

### O37. Barriers and enablers to accessing surveillance tests among childhood cancer survivors in Ontario, Canada

#### Jennifer Shuldiner^1^, Nida Shah^2^, Ann Marie Corrado^3^, Paul C. Nathan^2,4,5^, Noah Ivers^1,5,6^

##### ^1^Women’s College Hospital Institute for Health System Solutions and Virtual Care, Womens College Hospital, Toronto, Ontario, Canada; ^2^SickKids Research Institute, Toronto, Ontario, Canada; ^3^The Peter Gilgan Centre for Women’s Cancers, Womens College Hospital, Toronto, Ontario, Canada; ^4^Division of Hematology/Oncology, Hospital for Sick Children, Toronto, Ontario, Canada; ^5^Institute of Health Policy, Management and Evaluation, University of Toronto, Toronto, Ontario, Canada; ^6^Department of Family and Community Medicine, University of Toronto, Toronto, Ontario, Canada

###### **Correspondence:** Jennifer Shuldiner (Jennifer.shuldiner@wchospital.ca)

**Background:**

Survivors of childhood cancer require lifelong risk-tailored care to mitigate the risk of morbidity or premature mortality as a result of their prior cancer treatment (“late effects”). Despite evidence that surveillance focused on early detection of late effects improves health and reduces mortality, most adult survivors of childhood cancer do not complete recommended surveillance tests. We sought to elucidate the barriers and enablers to accessing evidence-based, high-yield surveillance tests among childhood cancer survivors.

**Method:**

This qualitative study involved one-on-one semi-structured interviews with adult survivors of childhood cancer (N=10). Participants were registered at the largest provincial cancer survivor program in Ontario, Canada, and eligible for the surveillance tests of interest but had not attended the clinic in over five years. We framed the interview guide and content analysis using the Theoretical Domains Framework, a tool specifically developed for implementation research to identify influences on desired behaviour.

**Results:**

Key barriers to completing recommended surveillance tests included a lack of knowledge regarding late effects, physical distance from specialised survivor services, and a lack of advice from family physicians. These barriers impacted the intention of survivors to obtain recommended surveillance tests or visit a speciliazed survivor clinic. Conversely, survivors discussed how they would not be deterred from getting a test if a physician recommended it, and those who had a health professional who referred survivors for tests were committed to obtaining them.

**Conclusion:**

Childhood cancer survivors prioritized their health and valued surveillance testing for late effects as a means to prevent illness. Poor awareness about the recommendations among survivors and their physicians must be addressed as a first step to implementation of guidelines. These findings will inform the planning and implementation of a centralized system to identify high-risk survivors and provide them and their physicians with personalized information about recommended surveillance.

### O38. A qualitative study to identify the factors influencing the perceived acceptability, appropriateness and feasibility of implementing a falls risk assessment service for older people

#### Susan Calnan, Caragh Flannery, Sheena McHugh

##### School of Public Health, University College Cork, Western Rd., Cork, Ireland

###### **Correspondence:** Susan Calnan (susan.calnan@ucc.ie)

**Background**

Falls are considered one of the most serious and common threats to older people’s ability to maintain their independence. In Ireland, a new integrated falls prevention pathway for older people was introduced in 2015, including multidisciplinary falls risk assessment clinics in primary care. The aim of this study is to identify the factors that influenced the acceptability, appropriateness and feasibility of implementation among those delivering the clinics.

**Methods**

Methods involved one-to-one interviews with healthcare professionals (physiotherapists, occupational therapists and nurses) delivering falls risk assessment clinics across four implementation sites. Interviews were conducted prior to implementation and six months after implementation had commenced, in 2016 and 2017. Data were analysed using a combination of the Consolidated Framework for Implementation Research (CFIR) and Proctor’s implementation outcomes taxonomy.

**Results**

The study identifies particular aspects of the implementation, as defined by CFIR, that influenced its acceptability, appropriateness and feasibility. Intervention characteristics, such as the relative advantages perceived and low complexity of the assessment clinics, positively influenced its perceived acceptability among service providers. Both outer setting (patient need for falls services) and inner setting (networks and communications) factors influenced its perceived appropriateness. Readiness for implementation, in particular the lack of available resources, strongly influenced the perceived feasibility of the service.

**Conclusion**

This study highlights the complex interplay between implementation outcomes. While an intervention may be deemed acceptable by service providers, for example, its perceived feasibility may be negatively impacted by practical constraints of the implementation setting. Results from this study will be used to improve future implementation of this complex health intervention and to inform the implementation of other falls prevention services for older people internationally.

### P39. Optimising the mining of electronic health records to implement health promotion apps via electronic messaging (OptiMine study)

#### Zarnie Khadjesari^1^, Tracey Brown^1^ Alex Ramsey^2^, Henry Goodfellow^3^, Sherine El-Toukhy^4^, Lorien Abroms^5^, Helena Jopling^6^, Michael Amato^7^

##### ^1^School of Health Sciences, University of East Anglia, Norwich, UK; ^2^Institute for Public Health, Washington University in St. Louis, St. Louis, Missouri, USA; ^3^Department of Primary Care and Population Health, University College London, London, UK; ^4^The National Institute on Minority Health and Health Disparities, The National Institutes of Health, Bethesda, Maryland, USA; ^5^Department of Prevention and Community Health, George Washington University, Washington DC, USA; ^6^West Suffolk NHS Foundation Trust, Bury St. Edmunds, Suffolk, UK; ^7^Truth Initiative, Washington DC, USA

###### **Correspondence:** Zarnie Khadjesari (z.khadjesari@uea.ac.uk)

**Background:**

Health promotion apps offer timely support, privacy and scalability, but are not currently delivered as part of routine patient care. Leveraging Electronic Health Records (EHR) to expand the reach of health promotion apps via electronic messaging provides a novel approach to implementation. We explored the acceptability, feasibility and reach of electronic messaging as an implementation strategy.

**Materials and methods:**

Three-phase, mixed-method study in an acute UK hospital. Public Health England apps were promoted: Smokefree and Drink Free Days. Phase 1: focus groups with patients and staff on the acceptability of sending electronic messages to patients (pre-implementation). Phase 2: feasibility of identifying at-risk behaviours and patient characteristics via EHR. Phase 3: reach of electronic messages to promote apps via an embedded link; primary outcome was the proportion of patients who click on the link. Online survey based on the Perceived Attributes of eHealth innovations explored acceptability of the electronic messages (post-implementation).

**Results:**

Six focus groups included 10 patients and 14 staff. SMS was the most suitable format for the messages, reinforced by analysis of the EHR data. Views on targeted vs. universal messaging were mixed (i.e. messages sent to at-risk patients vs. all patients), other pros and cons, technical complications and alternative resources were explored. 1526 patients were sent an SMS in January 2020, preliminary findings suggest 13% of patients accessed the link to the apps, which exceeded our 5% success rate. The online questionnaire was completed by 3.7%. Most participants were satisfied with the messages (80%), found them helpful (64%) and easy to comprehend (98%).

**Conclusions:**

SMS to promote uptake of health promotion apps to patients identified via the EHR is acceptable and feasible, which led to high reach. Our implementation strategy has the potential for reducing the burden of preventable death and disease at scale and low cost.

**Acknowledgements**

This study was funded by Cancer Research UK in collaboration with the National Cancer Institute (NCI).

### P40. OASI2: a hybrid effectiveness implementation RCT to inform scale up of care bundle to reduce obstetric anal sphincter injury (OASI) caused during childbirth

#### Magdalena Jurczuk^1^, Posy Bidwell^1^, Daniel Wolstenholme^1^, Louise Silverton^2^, Jan Van Der Meulen^3^, Nick Sevdalis^4§^, Ipek Gurol-Urganci^1,3§^, Ranee Thakar^5§^

##### ^1^Centre for Quality Improvement and Clinical Audit, Royal College of Obstetricians and Gynaecologists, London SE1 1SZ, UK; ^2^Royal College of Midwives, London W1G 9NH, UK; ^3^Department of Health Services Research and Policy, London School of Hygiene and Tropical Medicine, London WC1H 9SH, UK; ^4^Centre for Implementation Science, Health Service and Population Research Department, King’s College London, London SE5 8AF, UK; ^5^Croydon University Hospitals NHS Trust, Croydon CR7 7YE, UK

###### **Correspondence:** Magdalena Jurczuk (mjurczuk@rcog.org.uk)

^*§*^ *Authors Ranee Thakar, Nick Sevdalis and Ipek Gurol-Urganci contributed equally to the study and share senior authorship*

**Background**

Obstetric anal sphincter injuries (OASI) can have severe debilitating consequences for women. A large-scale quality improvement study (OASI1; 2017-18) implemented a care bundle (antenatal information to women, manual perineal protection and mediolateral episiotomy when indicated) in 16 maternity units in Britain [1] which reduced OASI rates. OASI2 (2021-22) is a scale-up study, which examines strategies used to introduce, implement and sustain implementation.

**Method**

OASI2 is a cluster-randomised control trial with two arms. Arm 1 (peer-to-peer implementation, n=10) is supported by peer units. Arm 2 (lean implementation, n=10) does not receive any active implementation support. A parallel nonrandomised study group (sustainability arm) consisting of original OASI1 units, allows study of the care bundle’s sustainability over time. An estimated 2,750 singleton live births/unit will be eligible for the care bundle. All three study groups receive an implementation toolkit including training resources. Table 1 details the expected implementation strategies across the three arms. Clinical outcomes (OASI rates) are collated from maternity information systems; implementation outcomes (acceptability, feasibility, appropriateness, sustainability) are collected through validated surveys [2,3] administered to women and clinicians, supplemented by qualitative research. Quantitative data are analysed using regression modelling and descriptive statistics.

**Results**

The trial will identify the effect of the applied implementation strategies [4] on implementation success, and link that to the clinical effectiveness of the bundle. Successful sustainability strategies will be identified.

**Conclusion**

The study will generate insights into how to effectively scale-up and sustain uptake and coverage of similar interventions in maternity units. A locally adaptable ‘implementation blueprint’, will be produced to inform development of future guidelines to prevent perineal trauma.

**Acknowledgements**

The study is funded by the Health Foundation. NS’s research is supported by the NIHR Applied Research Collaboration South London. We are also grateful to our independent advisory group who have contributed to the conceptualization, design and implementation of both the OASI1 and the OASI2 studies.

**Trial Registration**

Trial registration pending completion of research protocol.

**References**

1. Bidwell P, Thakar R, Sevdalis N, Silverton L, Novis V, Hellyer A, et al. A multi-centre quality improvement project to reduce the incidence of obstetric anal sphincter injury (OASI): Study protocol. BMC Pregnancy Childbirth. 2018;18(1):1–33.

2. Weiner BJ, Lewis CC, Stanick C, Powell BJ, Dorsey CN, Clary AS, et al. Psychometric assessment of three newly developed implementation outcome measures. Implement Sci. 2017;12(1):1–12.

3. University W. Clinical Assessment Sustainability Tool. 2012.

4. Powell BJ, Waltz TJ, Chinman MJ, Damschroder LJ, Smith JL, Matthieu MM, et al. A refined compilation of implementation strategies: Results from the Expert Recommendations for Implementing Change (ERIC) project. Implement Sci. 2015;10(1):1–14.

**Table 1 (abstract P40).** Discrete implementation strategies to be used in each group of participating units

Discrete Implementation Strategy/ group of strategies [4]		Sustainability group	Peer implementation	Lean implementation
Implementation requirements	Identify and prepare champions/ Facilitation/ Clinical supervision	x	x	x
	Develop and organize quality monitoring systems/ change record systems/ Audit & provide feedback / facilitate relay of clinical data to providers	x	x	x
	Develop a formal implementation blueprint/ develop an implementation glossary	x	x	x
	Conduct educational meetings (clinicians)	x	x	x
	Use educational materials (toolkit resources)	x	x	x
	Remind clinicians: pens, posters, stickers (care bundle launch day and promote the care bundle continuously)	x	x	x
	Obtain and use patient feedback/ Prepare patients/consumers to be active participants (engage local PPI group)	x	x	x
Strategies exclusive to external facilitation	Centralise technical assistance/ Provide local technical assistance/ Use an implementation advisor/ Provide ongoing consultation	Centralised technical assistance	Local technical assistance	
	Organize clinician implementation team meetings (skills development days led by external facilitators)	x	x	
	Create a learning collaborative/ Promote network weaving	x	x	
	Use train the trainer strategies	Project Team > clinicians through cascade	External facilitators > clinicians through cascade	
Strategies related to sustainability efforts	Conduct educational outreach visits	Site visits from Project Team		
	Involve executive boards	Ensure senior-buy in		
	Mandate change/ Create or change credentialing and/or licensure standards	Bundle introduced into mandatory training/ induction packages		
	Fund & contract for the clinical innovation	Protected time or champions		
	Revise professional roles	Formal titles for champions		
	Recruit, designate, train for leadership	Champions trained for external facilitator role		

### P41. Healthcare stakeholders’ perceptions and experiences of factors affecting the implementation of critical care telemedicine (CCT): qualitative evidence synthesis

#### Andreas Xyrichis, Katerina Iliopoulou

##### King’s College, London, Strand, WC2R 2LS, UK

###### **Correspondence:** Andreas Xyrichis (andreas.xyrichis@kcl.ac.uk)

**Background:**

Critical care telemedicine (CCT) has long been used to expand the delivery of best care to critically ill patients located in geographically distant areas [1]. During the COVID-19 pandemic those health systems with CCT appeared better prepared to respond to the pandemic [2]. However, several challenges remain for CCT to be effectively implemented [3]. This review synthesises qualitative evidence on healthcare stakeholders’ perceptions and experiences of factors affecting implementation of CCT, with a view to developing hypotheses about factors more likely to foster successful implementation.

**Method:**

We systematically searched five databases for empirical qualitative studies published in any language. The search combined terms for telemedicine with critical care, decision support, and remote monitoring. We independently screened the reference lists of included studies and searched five sources for grey literature. Two reviewers extracted data and appraised included studies independently and in duplicate. Conflicts were resolved in the team. We used the CFIR[4] to inform data synthesis. Additional themes not captured by CFIR were classified under a separate theme. We used GRADE-CERQual [5] to assess our confidence in the findings.

**Results:**

Thirteen studies were included representing a range of settings but all from North America. We identified 20 review findings that affect implementation of CCT. The majority of factors mapped to three CFIR domains: intervention characteristics, inner setting, and characteristics of individuals. Factors relating to networks and communication, along with interactions between hub and bedside teams, were the most prominent review findings

**Conclusion:**

We have high or moderate confidence in the evidence contributing to several of the review findings. Further qualitative research, especially in contexts other than North America, which are subject to different social and cultural values, would strengthen the evidence base. Future implementation research is needed to build on our findings and examine appropriate strategies for further implementation of CCT.

**Acknowledgements**

This review is being completed with the contribution of Dr Mackintosh NJ, Dr Terblanche M, Professor Bench S, Dr Philippou J, Professor Sandall J

**References**

1. Xyrichis A, Mackintosh NJ, Terblanche M, Bench S, Philippou J, Sandall J. Healthcare stakeholders’ perceptions and experiences of factors affecting the implementation of critical care telemedicine (CCT): qualitative evidence synthesis. COCHRANE DB SYST REV. 2017; 11.

2. Ohannessian R, Duong TA, Odone A. Global telemedicine implementation and integration within health systems to fight the COVID-19 pandemic: a call to action. JMIR public health and surveillance. 2020; 6(2): e18810.

3. Mackintosh N, Terblanche M, Maharaj R, Xyrichis A, Franklin K, Keddie J, Larkins E, Maslen A, Skinner J, Newman S, Magalhaes JH. Telemedicine with clinical decision support for critical care: a systematic review. Syst Rev. 2016; 5 (1):176.

4. Damschroder LJ, Aron DC, Keith RE, Kirsh SR, Alexander JA, Lowery JC. Fostering implementation of health services research findings into practice: a consolidated framework for advancing Implementation Sci. Implementation Sci. 2009; 4(1):50.

5. Noyes J, Booth A, Lewin S, Carlsen B, Glenton C, Colvin CJ, Garside R, Bohren MA, Rashidian A, Wainwright M, Tunςalp Ö. Applying GRADE-CERQual to qualitative evidence synthesis findings–paper 6: how to assess relevance of the data. Implementation Sci. 2018 Jan;13(1):4.

### P42. Improving outcomes for children with communication difficulties: Implementing cross-disciplinary service delivery models in schools

#### Jessica McCluskey^1 2^, Patricia Donnelly^1 2^, Sarah Brady^1 2^, Sue Franklin^1 2^, Carol-Anne Murphy^1 2^

##### ^1^School of Allied Health, University of Limerick, Limerick, Ireland; ^2^ Health Implementation Science and Technology Cluster, Health Research Institute, (HIST_HRI) University of Limerick, Ireland

###### **Correspondence:** Carol-Anne Murphy (carol-anne.murphy@ul.ie)

**Background:**

Children and adolescents with speech, language and communication needs (SLCN) comprise approximately 10% of the school population and are at risk of adverse social, emotional, academic and vocational outcomes. Providing effective in-school language supports requires feedback and coaching involving speech and language therapist (SLT)/teacher collaboration [1, 2, 3]. We describe a model of SLT/teacher support for students with SLCN, using the Re-Aim framework [4].

**Method:**

Tailored CPD was offered to 4 schools, followed by coaching; and co-teaching (SLTs and Special Education teachers) of 10 groups of children with SLCN.

**Results:**

Re-Aim [4] objectives were achieved as follows

*Reach****:*** we identified key school leaders and contextualised the proposed work within school improvement plans (e.g. linking the SLCN programme to literacy and behaviour)

*Effectiveness****:*** SLTs supported teachers to monitor programme implementation, fidelity and success; we gathered data on key stakeholder (SLTs, teachers, students and parents) perspectives

*Adoption:* we attended schools’ special education planning meetings (2) had regular contact with school leadership (3) provided a flexible support model tailored to the individual school context.

*Implementation:* as well as co-teaching, SLTs and teachers engaged in reflective collaborative meetings regarding delivery

*Maintenance* of the intervention was achieved through tailored whole school CPD workshops and building capacity of teachers involved to advocate for the impact of interventions and strategies on students and teaching practice

Intervention delivery outcomes and key stakeholder perspectives, informed the development of a revised and updated model of collaborative working. Students clearly benefitted from the language support, and key personnel have increased capacity for sustained collaboration.

**Conclusion:**

The revised model of working, derived from best practice regarding inter-professional collaboration and school capacity building, is enhanced by knowledge gained through implementation in a local context. Given national and international policies to embed therapy in the education context, this can inform cross-sectoral collaboration initiatives.

**Acknowledgements**

This work is being conducted through a tender (RFT270618)

awarded by the National Council for Special Education (NCSE) in Ireland, to University of Limerick for the Provision of Speech and Language Therapy Support Services to Schools.

**References**

1. Starling, J., Munro, N., Togher, L. & Arcuili, J. Training Secondary School Teachers in Instructional Language Modification Techniques to Support Adolescents with Language Impairment: A Randomized Controlled Trial. Lang, Speech, Hear Serv School, 2012; 43: 474-495. 10.1044/0161-1461(2012/11-0066)

2. Snow, P.C, Eadie, P.A, Connell, J., Dalheim, B., McCusker, H.J & Munro, J.K Oral language supports early literacy: A pilot cluster randomized trial in disadvantaged schools, Int J Speech Lang Pathol, 2019; 16:5: 495-506, 10.3109/17549507.2013.845691

3. Ebbels, S., McCartney, E., Slonims, V., Dockrell, J., & Norbury, C. Evidence-based pathways to intervention for children with language disorders. Int J Lang Commun Disord, 2019; 54:1: 3-19 10.1111/1460-6984.12387

4. Glasgow RE, Vogt TM, Boles SM: Evaluating the public health impact of health promotion interventions: The RE-AIM framework. Am J Public Health 1999; 89:1322–1327.

### O43. Key findings from an outcomes evaluation of a ‘scaled up’ domestic abuse recovery programme

#### Emma Smith, Emma Belton

##### National Society for the Prevention of Cruelty to Children, London, UK

###### **Correspondence:** Emma Smith (Emma.smith@NSPCC.org.uk)

**Background**

As part of the NSPCC 2016-2021 strategy, the charity scaled up a number of its evidence-based programmes in order that more children could potentially benefit. This included Domestic Abuse Recovering Together (DART), a programme designed to support mothers and children through their recovery from domestic abuse. DART is currently implemented in 28 UK sites, including local authorities and voluntary organisations.

Following an implementation evaluation, an outcomes evaluation was conducted to see whether or not non-NSPCC services, supported by the NSPCC to deliver DART, could achieve similar outcomes for service users as evidenced by the evaluation of the original service.

**Method**

A quasi experimental design involved an intervention group (comprised of families from six scale up sites), a no intervention group (Families from three NSPCC sites waiting to attend DART) and evaluation data from the original NSPCC DART services. The same standardised measures were completed by all sites at two time points.

**Results**

Independent samples t tests revealed significant improvements in all outcomes measured pre and post DART for scale up sites: Mothers had significantly greater self-esteem, an improved relationship with their child and their child had fewer emotional and behavioural difficulties. These improvements were significantly greater than the no-intervention group, but very similar to the original DART groups run by the NSPCC.

**Conclusion**

The results suggested that external organisations were equally as successful as the NSPCC at implementing DART, with very similar positive outcomes for families.

### P44. The practical application of a technology implementation framework to the concurrent development of a medical device and a new clinical service: Custom-made 3D printed Non-Invasive Ventilation masks for paediatric patients

#### Katherine Jeays-Ward^1^, Matt Willox^2^, Nicki Barker^3^, Pete Metherall^4^, Avril McCarthy^1,5^, Heath Read^2^, and Heather Elphick^3^

##### ^1^NIHR Devices for Dignity Med Tech Co-operative, Sheffield Teaching Hospitals NHS Foundation Trust, Sheffield, UK; ^2^ACES, Sheffield Hallam University, Sheffield, UK; ^3^Sheffield Children’s NHS Foundation Trust, Sheffield, UK; ^4^3-D Lab, Sheffield Teaching Hospitals NHS Foundation Trust, Sheffield, UK; ^5^Clinical Engineering, Sheffield Teaching Hospitals NHS Foundation Trust, Sheffield, UK

###### **Correspondence:** Katherine Jeays-Ward (Katherine.jeays-ward@nihr.ac.uk)

**Background**

Non-invasive ventilation (NIV) is assisted mechanical ventilation delivered via facemask for people with chronic respiratory conditions. Masks that fit well are difficult to find for children who have small or asymmetrical facial features. We are addressing the need for improved paediatric NIV masks by concurrently developing bespoke 3D printed masks and a new clinical service to provide them.

The Non-adoption, Abandonment and challenges to Scale-up, Spread and Sustainability (NASSS) implementation framework [1] is a technology-specific framework that acknowledges the problematic system complexity associated with sustainable implementation of technology-supported changes in healthcare.

We present the application of NASSS framework principles to proactively address barriers to sustainable implementation during device and service development.

**Method**

The adaptable NASSS framework includes domains specific to Adopters and Technology. Priority setting events took place with patients and professionals, and detailed assessment of suitable materials and manufacturing methods. We performed breakdowns of potential service pathways, identifying where and how individuals would interact with the service and devices, and challenging the pathway with different eventualities. This was undertaken iteratively using co-production and formative evaluation methods to concurrently guide both product and service development.

**Results**

We worked with multiple stakeholder groups to identify priorities for product development and aspects of care. Priorities included processes for identification of suitable patients, prescriptions, mask comfort, and medical device interoperability. Barriers that were identified and resolved included patient access to 3D scanning facilities, quality of scan data, and regulatory standards.

**Conclusion**

By developing a feasible clinical pathway [2] we have bypassed multiple potential pitfalls to eventual adoption into practice. A clinical trial of the resultant customised masks is currently underway.

This need-driven project prioritised implementation from the outset. It combined iterative product development and concurrent service creation, and is a de-risking approach to NHS-led technology innovation that can be replicated by other medtech developments.

**Acknowledgements**

This research is funded by the National Institute for Health Research (Invention for Innovation, i4i; II-LB-0814-20004). The views expressed are those of the author and not necessarily those of the NHS, the National Institute for Health Research or the Department of Health and Social Care.

**References**

^1^ Greenhalgh T, Abimbola S. The NASSS Framework - A Synthesis of Multiple Theories of Technology Implementation. Stud Health Technol Inform. 2019; 263:193-204.

^2^ Willox M, Metherall P, Jeays-Ward K, McCarthy A, Barker N, Reed H, Elphick H. Custom-made 3D printed masks for children using non-invasive ventilation: a feasibility study of production method and testing of outcomes in adult volunteers. J Med Eng Technol. 2020; Jun 29:1-11.

